# Out of Arabia: A Complex Biogeographic History of Multiple Vicariance and Dispersal Events in the Gecko Genus *Hemidactylus* (Reptilia: Gekkonidae)

**DOI:** 10.1371/journal.pone.0064018

**Published:** 2013-05-27

**Authors:** Jiří Šmíd, Salvador Carranza, Lukáš Kratochvíl, Václav Gvoždík, Abdul Karim Nasher, Jiří Moravec

**Affiliations:** 1 Department of Zoology, National Museum, Prague, Czech Republic; 2 Department of Zoology, Faculty of Science, Charles University in Prague, Prague, Czech Republic; 3 Institute of Evolutionary Biology (CSIC-UPF), Barcelona, Spain; 4 Department of Ecology, Faculty of Science, Charles University in Prague, Prague, Czech Republic; 5 Department of Environmental Sciences, Biogeography, University of Basel, Basel, Switzerland; 6 Faculty of Science, University of Sana'a, Sana'a, Yemen; George Washington University, United States of America

## Abstract

The geological history of the Arabian Peninsula has played a crucial role in shaping current diversity and distribution patterns of many Arabian and African faunal elements. The gecko genus *Hemidactylus* is not an exception. In this study, we provide an insight into the phylogeny and systematics of 45 recognized species of the so-called Arid clade of the genus *Hemidactylus* from Arabia, the Horn of Africa, the Levant and Iran. The material comprises 358 specimens sequenced for up to two mitochondrial (12S rRNA, cytochrome *b*) and four nuclear (*mc1r*, *cmos*, *rag1*, *rag2*) genes with 4766 bp of the concatenated alignment length. A robust calibrated phylogeny and reconstruction of historical biogeography are inferred. We link the history of this genus with major geological events that occurred in the region within the last 30 million years. Two basal divergences correspond with the break-ups of the Arabian and African landmasses and subsequent separation of Socotra from the Arabian mainland, respectively, segregating the genus by means of vicariance. Formation of the Red Sea led to isolation and subsequent radiation in the Arabian Peninsula, which was followed by multiple independent expansions: 13.1 Ma to Iran; 9.8 Ma to NE Africa; 8.2 to Socotra Archipelago; 7–7.3 Ma two colonizations to the Near East; 5.9 Ma to NE Africa; and 4.1 to Socotra. Moreover, using multiple genetic markers we detected cryptic diversity within the genus, particularly in south-western Arabia and the Ethiopian highlands, and confirmed the existence of at least seven new species in the area. These findings highlight the role of Arabia and the Horn of Africa as an important *Hemidactylus* diversity hotspot.

## Introduction

With 122 currently valid species, the genus *Hemidactylus* is the second most speciose gecko genus (after *Cyrtodactylus*) and ranks among the top ten species-rich genera of reptiles [1]. *Hemidactylus* geckos are widely distributed across all tropical and subtropical continental landmasses and hundreds of intervening continental and oceanic islands, from Southeast Asia westwards over Africa to the New World [2], [3]. As already shown by many authors [4]–[10], the current distribution of the genus has been highly affected by repeated transmarine colonizations caused either by human activity or by spontaneous rafting, which have contributed significantly to the unusually wide distribution range of the genus [4], [6]. For instance, the transatlantic colonization of Central and South America by African species has occurred independently at least four times [10]. The most species-rich areas include the tropics and subtropics of the Old World, with the highest species richness being achieved in the Horn of Africa (Somalia and adjacent countries), which, based on the current taxonomy of the genus, is known to be inhabited by 38 species [1], [11]–[13].

As a result of its wide distribution and high species richness, the genus *Hemidactylus* represents an excellent model for testing biogeographic, ecological and evolutionary hypotheses, and has therefore become a centre of attention of molecular phylogenetic studies [5]–[7], [14]–[26]. The first comprehensive phylogeny covering about one third of all *Hemidactylus* species was published by Carranza and Arnold [6]. Taking into account additional adjustments [19], [23], this work resulted in the division of the genus into four phylogenetic lineages: (i) Tropical Asian clade, (ii) *H. angulatus* clade, (iii) Arid clade, and (iv) African – Atlantic clade. All Arabian *Hemidactylus* species belong to the Arid clade with only two exceptions: *H. flaviviridis* and *H. leschenaulti,* which are part of the Tropical Asian clade and have most probably been introduced into Arabia by human-mediated transportation [7]. Mainland Arabian *Hemidactylus* have witnessed a substantial increase of described taxa, from 9 to 21 within the last two years [7], [21], [23]. Moreover, recent works from the Levant [23] and the Socotra Archipelago [26] reported the occurrence of several unnamed (or putative) species in the Sinai, Yemen mainland and Socotra, suggesting that the real diversity of the Arabian members of the Arid clade of *Hemidactylus* is still largely underestimated. In contrast to the relatively high number of recent studies on Arabian *Hemidactylus*, virtually nothing is known about Northeast African *Hemidactylus* from a phylogenetic point of view. Preliminary analyses including up to 9 *Hemidactylus* species [6], [7] suggest that these belong to the Arid clade, the *H. angulatus* clade or the African-Atlantic clade. The main reason of the poor knowledge of Northeast African *Hemidactylus* is the difficult accessibility of the region, which has made it almost impossible to perform any systematic zoological research for the last two decades.

Looking at the region from a geological perspective, the process of separation of the Arabian Peninsula from the African landmass took place from the mid-Oligocene to the Early Miocene (31–23 Ma) as a consequence of the East African Rift system faulting, which resulted in the formation of the Red Sea and the Gulf of Aden [27]–[29]. This continental break-up propagated from East to West, splitting the oceanic crust and triggering the separation of the Socotra Archipelago from the Dhofar region in Oman approximately 24 Ma [29]–[31]. Africa and Arabia became reconnected in a period between 10–5.3 Ma when massive halite deposits formed a land bridge in the Bab-el-Mandeb strait [29], [32]; for general map with geographic names used in the text see Fig. S1. The long-term connectivity between Africa and Arabia and the subsequent geological events have had a crucial impact on the regional biogeography and explain the close biogeographic affinities between NE African and SW Arabian faunas [33], [34]. It has been suggested that the diversity and distribution of current Afro-Arabian herpetofauna was influenced mainly by the three following factors: 1) the formation of the Red Sea in the Oligocene (27–24 Ma), which resulted in a vicariance event separating African and Arabian fauna [35], [36]; 2) temporary reconnection of Africa and Arabia 10–5.3 Ma [29] and the geographic proximity of these landmasses, particularly in the narrowest point (Bab-el-Mandeb strait), which was only 5 km wide during the driest periods within the last 0.5 million years [37] and may have facilitated faunal exchange by means of dispersal [32], [34]; and 3) the penetration of some Afrotropical and Mediterranean elements to SW Arabia from the north along the Hijaz and Asir mountain ranges, which provide suitable conditions for more temperate species than the otherwise arid desert environment of the Arabian Peninsula [35], [38], [39]. All these factors may have affected speciation and current distribution of *Hemidactylus* geckos.

In the present study, we provide new molecular data for *Hemidactylus* geckos from Arabia and the Horn of Africa and produce the most complete phylogeny to date of *Hemidactylus* from the Arid clade with the intention to: (1) evaluate the phylogenetic relationships among individual *Hemidactylus* populations and assess their systematics, (2) increment our knowledge on the *Hemidactylus* species from Arabia and the Horn of Africa and assess their mutual affinities, (3) reanalyze recent patterns of geographic distribution and reconstruct potential ways of historical dispersal routes or vicariance events, and (4) find possible correspondences between the geological history of the region with evolutionary splits of ancestral lineages in *Hemidactylus*.

## Methods

### Ethic Statement

Most of the investigated material comes from museum voucher specimens (BMNH London, CAS San Francisco, IBE Barcelona, NMP Prague; see Table S1). Vouchers and tissue samples were kindly accessed as loans by the appropriate curators with their permission to use the samples for DNA analyses (B. Clarke and E. N. Arnold – BMNH; J. Vindum – CAS; S. Carranza – IBE; J. Moravec – NMP). Remaining samples were obtained in the field with appropriate collecting permits (Oman: issued by Ali Alkiyumii, Ministry of Environment and Climate Affairs of the Sultanate of Oman: Refs 08/2005, 16/2008, 38/2010, 12/2011; Yemen: issued by Omer Baeshen, Environment Protection Agency, Sana'a, Republic of Yemen: Ref 10/2007; Kenya: issued by National Council for Science and Technology (NCST), Nairobi, Kenya). No endangered or protected species was collected and no samples from protected or private areas were used for this study. Research was conducted with the approval of Central Commission for Animal Welfare, the Czech Republic, accreditation no. 1090/2012–MZE–17214. All efforts were made to minimize animal suffering.

### Tissue Samples, DNA Extraction and PCR Amplification

In total, sequences of 358 *Hemidactylus* specimens were used in this study. Additionally, 15 sequences of the 12S rRNA (*12S*) mitochondrial gene of three taxa recently described from Yemen [21], which were kindly donated by U. Joger, were included into the analysis. Ten specimens of *H. flaviviridis* were used as outgroups [7]. Localities, specimen codes and GenBank accession numbers of all genes included in the phylogenetic analyses are shown in Table S1.

Total genomic DNA was extracted using Geneaid Extraction Kit and DNeasy Tissue Kit (Qiagen) following the protocols therein. Two mtDNA genes (partial sequence of *12S*, and cytochrome *b* - *cytb* ) and four nDNA genes encoding the proto-oncogene *mos* (*cmos*), the melano-cortin 1 receptor (*mc1r*) and the recombination activating genes 1 and 2 (*rag1* and *rag2*, respectively) were amplified. Two sets of primers were used for the *cytb*: one set for the complete 1137 bp of the *cytb* gene and, when this long fragment failed to amplify, a second set that amplifies a shorter region of 307 bp was employed [6], [7]. Also for *rag1*, two pairs of primers were used: one for a region of over 1000 bp and, when as a result of poor DNA quality this long fragment could not be amplified, a second pair of primers amplifying 280 bp was employed. A complete list of all primers used, their sequences, length of amplified region, PCR conditions and source is given in Table S2.

### Sequence Alignment

Apart from the genes amplified for the present study (see above), the final alignment included also the mitochondrial NADH dehydrogenase 4 (*nd4*) coding gene and the adjacent tRNA region (*tRNAs*; including the complete sequences of tRNA-His and tRNA-Ser and the first eight nucleotides of tRNA-Leu) from [7]. Chromatograms of all sequences newly produced for this study were checked by eye and assembled using the software Geneious v. 5.3.6 [40]. DNA sequences were aligned using MAFFT v.6 [41] with the options maxiterate 1000 and localpair. Poorly aligned positions of some mtDNA regions (*12S* and *tRNAs*) were eliminated with G-blocks [42] using low stringency options [43]. No stop codons were detected after translation of the protein-coding genes with standard genetic code for nuclear genes and the vertebrate mitochondrial code for the *cytb* and *nd4* genes into amino acids, suggesting that all genes are functional and no pseudogenes were amplified. Occasional heterozygous positions in the nuclear genes were coded according to the IUPAC ambiguity codes.

### Phylogenetic Analyses

The final alignment of all concatenated genes included 4766 bp (2267 bp of mtDNA and 2499 bp of nDNA). The best-fitting model of nucleotide substitution was assessed for each gene independently using jModelTest v.0.1.1 [44] under the Akaike information criterion (AIC). All information related to each partition including alignment length, model selected, and the number of variable and parsimony-informative sites are presented in Table 1.

**Table 1:** Summary of DNA partitions.

Gene	Length (bp)	Model	Var	Pars. inf.	LRT
*cytb*	295–1137	GTR+G	615	550	not rejected (*P*<0.47086)
*nd4*	588	GTR+I+G	314	252	rejected (*P*<0.00037)
*tRNAs*	146	GTR+G	75	58	rejected (*P*<0.00424)
*12S*	317–396	GTR+I+G	200	167	rejected (*P*<5.05957E-9)
*cmos*	402	TPM1+I+G	59	36	not rejected (*P*<0.15766)
*mc1r*	666	GTR+I+G	99	73	not rejected (*P*<0.08567)
*rag1*	280, 1023	GTR+G	138	75	not rejected (*P*<0.52772)
*rag2*	408	TrN+I+G	60	39	rejected (*P*<0.00475)

Information on the length of all partitions used in the phylogenetic analyses, model of sequence evolution selected by jModelTest [44] (Model), number of variable (Var) and parsimony-informative (Pars. inf.) sites calculated for the ingroup only, and the results of the test of rate homogeneity (LRT) run in MEGA [52] using only the subset of 58 sequences selected for the BEAST analysis (see Methods).

Maximum Likelihood (ML) and Bayesian Inference (BI) analyses were performed to infer the phylogenetic relationships among the taxa included in the present study (Tab. S1). ML analyses were performed in RAxML v 7.0.3 [45] with a GTR+I+G model of evolution with 100 random addition replicates and partition branch lengths and parameters estimated independently for each partition. Nodal support of the ML tree was assessed by 1000 bootstrap pseudoreplications [46]. Bayesian analyses were performed in MrBayes 3.1.2 [47] with appropriate best fitting models applied to all partitions (Tab. 1) and all parameters unlinked across partitions. Analyses were run for 10^7^ generations with sampling frequency of 1000 generations. After assurance that the log-likelihood achieved stationarity (as plotted against generations), the first 20% of obtained trees were discarded as a burn-in and a 50% majority rule consensus tree was produced from the posterior distribution of the trees and posterior probabilities calculated as the percentage of a sampled tree recovering any particular clade [48]. Nodes that received ML bootstrap support values ≥70% and posterior probability (pp) values ≥0.95 were considered strongly supported [48], [49]. To filter out the potentially strong bias of mtDNA on the resulting phylogeny, another dataset containing nuclear genes (unphased) only was assembled and used for the same phylogenetic analyses (ML, BI) with the same settings as described above and the results were compared with that of mtDNA+nDNA analyses.

### Molecular Dating Analysis

As already highlighted [7], the lack of internal calibration points in *Hemidactylus* precludes the direct estimation of the time of the cladogenetic events in our phylogeny. Therefore, the mean substitution rate of the same *cytb* and *12S* mitochondrial regions calculated for other lizard groups [7] was used for this purpose. Specifically, we set a normal distribution prior for the ucld.mean parameter of the *12S* and *cytb* partitions based on the combined meanRate posteriors (mean ± standard error) (0.00755±0.00247 for *12S* and 0.0228±0.00806 for *cytb*). The dataset for molecular dating analysis comprised sequences from all eight partitions (see Tab. 1; all nuclear genes unphased) from which the substitution rates of the *12S* and *cytb* partitions were used to estimate dates of the cladogenetic events. The analysis was performed in BEAST v. 1.6.1 [50]. As is customary for such analyses, we used a phylogeny pruned arbitrarily to include one representative from each of the major lineages uncovered with the concatenated analysis (58 specimens in total; see Tab. S1). This method excludes closely related terminal taxa because the Yule tree prior does not include a model of coalescence, which can complicate rate estimation for closely related sequences [51]. A likelihood-ratio test implemented in MEGA 5 [52] was used to test if the different partitions included in the dating analysis were evolving clock-like (see Tab. 1). This information was used to choose between the strict-clock and the relaxed uncorrelated lognormal clock priors implemented in BEAST [53]. Analyses were run four times for 5x10^7^ generations with a sampling frequency of 10 000. Models and prior specifications applied were as follows (otherwise by default): GTR+I+G, strict clock (*mc1r*, *cmos*); GTR+G, strict clock (*rag1*, *cytb*); GTR+I+G, relaxed uncorrelated lognormal clock (*nd4*, *12S*), GTR+G, relaxed uncorrelated lognormal clock (*tRNAs*); TrN+I+G, relaxed uncorrelated lognormal clock (*rag2*); Yule process of speciation; random starting tree; alpha Uniform (0, 10); yule.birthRate (0, 1000); ucld.mean of *12S* Normal (initial value: 0.00755, mean: 0.00755, Stdev: 0.00247); ucld.mean of *cytb* Normal (initial value: 0.0228, mean: 0.0228, Stdev: 0.00806).

### Biogeographic Analysis

**Figure 1:**
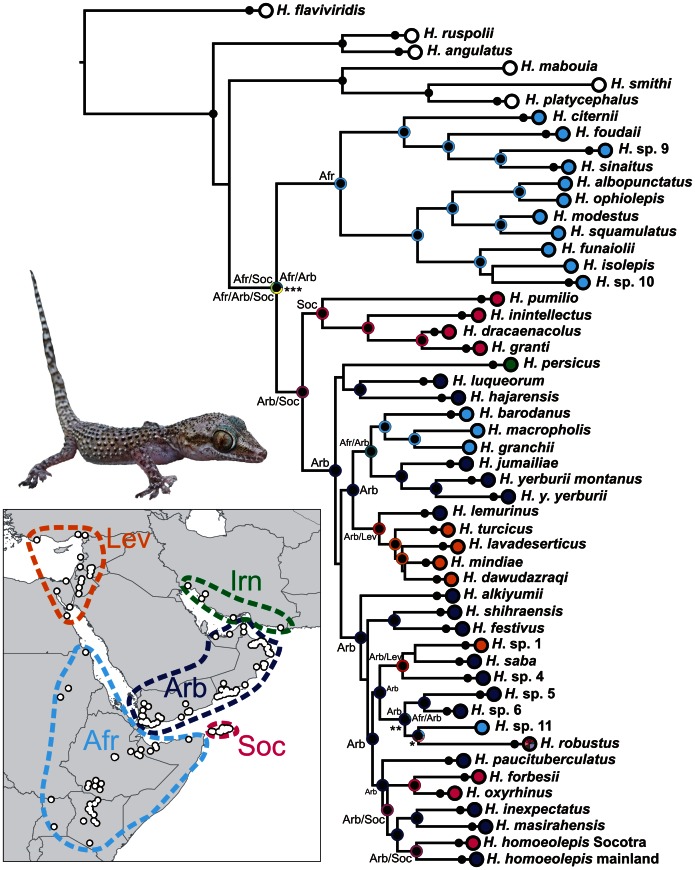
Maximum likelihood phylogenetic tree of the Arid clade of the genus *Hemidactylus*. Individuals of one species are collapsed into one terminal branch. Black dots on the nodes and on the terminal lineages indicate ML bootstrap values ≥70 and BI posterior probabilities ≥0.95. Species are coloured according to their geographic origin marked on the inset map where the sampling is also depicted. Colours and abbreviations in the nodes indicate reconstruction of ancestral distribution. The probability of the ancestral area reconstruction of the node marked with * is: Afr 19%, Afr/Arb 33.3%, Afr/Arb/Lev 19%, Afr/Arb/Irn 19%, Afr/Arb/Lev/Irn 9.5%; of the node **: Arb 80.5%, Afr/Arb 19.5%; of the node ***: Afr/Arb 33.3%, Afr/Soc 33.3%, Afr/Arb/Soc 33.3%; for all other nodes 100% for the area depicted. Undescribed species are labelled in accordance with previous works [7.23].

To reconstruct the biogeographic history of the Arid clade *Hemidactylus* species included in our phylogenetic analyses we used S-DIVA 1.9b [54], a statistical extension of the dispersal-vicariance analysis DIVA [55]. S-DIVA employs all sampled trees, not only the final consensus phylogeny, to reconstruct ancestral states and weights the ancestral distribution reconstruction at each node by the frequency of the given node. The same dataset used for the molecular dating analysis, containing 58 specimens, was employed for the biogeographic analysis. A BI analysis with the same settings as was used to infer the BI tree of the complete dataset was run (see above). The resulting 10 000 trees were imported into S-DIVA and the burn-in was performed therein. Species were assigned to five separated and well-defined geographic areas (Fig. 1): 1) Horn of Africa, including parts of NE Sudan; 2) South Arabia, consisting of Yemen, Oman, and United Arab Emirates; 3) Socotra Archipelago; 4) Levant and Sinai; and 5) Iran. In the widely distributed *H. robustus*, multiple geographic areas were defined according to the origin of our samples. The outgroup species were not evaluated in this analysis. The maximum number of unit areas allowed in the ancestral distribution (“Max areas”) was constrained to 4 and the “Allow reconstruction” option was activated. All other settings were left by default.

## Results

**Figure 2:**
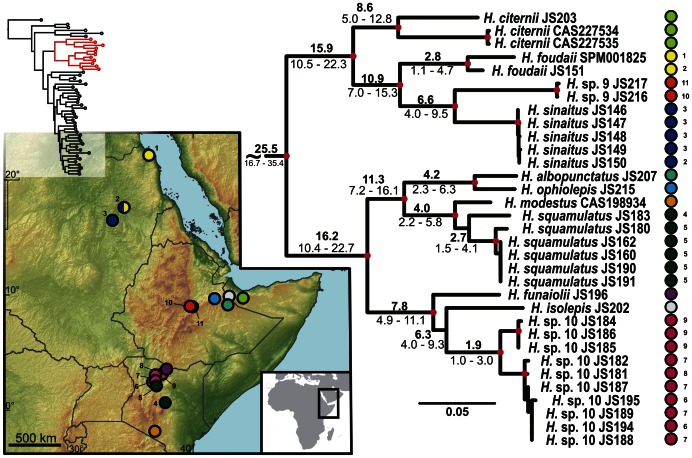
Detail of the phylogenetic tree of the Arid clade *Hemidactylus*: African subclade. **Red dots in the nodes indicate ML bootstrap values ≥70 and BI pp≥0.95.** **** Numbers after species names refer to sample codes; numbers on the right correspond with the localities numbers in the map. Ages of the nodes estimated with BEAST dating analysis are indicated by the nodes, mean above in bold, 95% HPD interval in plain below.

**Figure 3:**
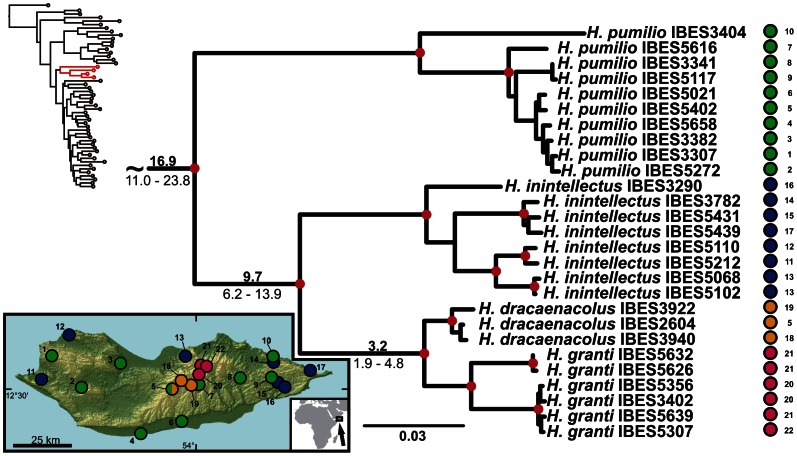
Detail of the phylogenetic tree of the Arid clade *Hemidactylus*: Socotran subclade. Red dots in the nodes indicate ML bootstrap values ≥70 and BI pp≥0.95. Numbers after species names refer to sample codes; numbers on the right correspond with the localities numbers in the map. Ages of the nodes estimated with BEAST dating analysis are indicated by the nodes, mean above in bold, 95% HPD interval in plain below.

**Figure 4:**
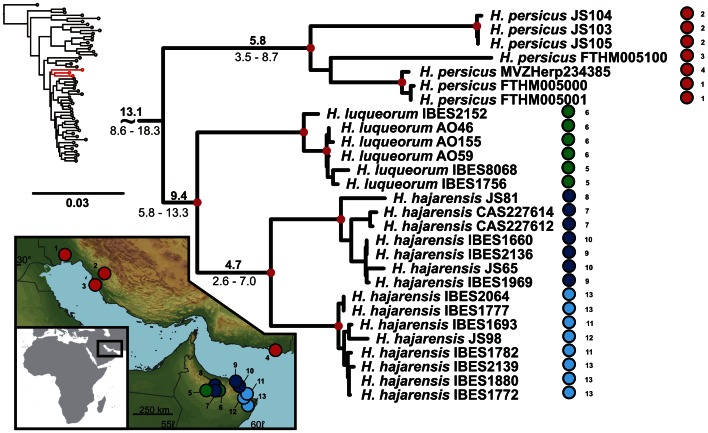
Detail of the phylogenetic tree of the Arid clade *Hemidactylus*: The Persian Gulf. Red dots in the nodes indicate ML bootstrap values ≥70 and BI pp≥0.95. Numbers after species names refer to sample codes; numbers on the right correspond with the localities numbers in the map. Ages of the nodes estimated with BEAST dating analysis are indicated by the nodes, mean above in bold, 95% HPD interval in plain below.

**Figure 5:**
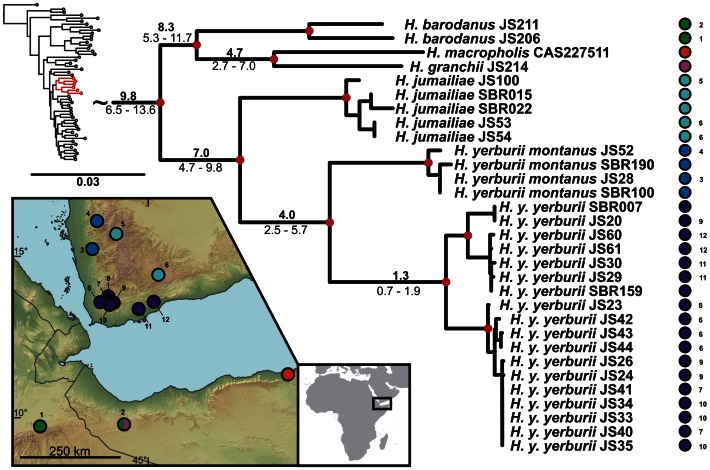
Detail of the phylogenetic tree of the Arid clade *Hemidactylus*: The Gulf of Aden. Red dots in the nodes indicate ML bootstrap values ≥70 and BI pp≥0.95. Numbers after species names refer to sample codes; numbers on the right correspond with the localities numbers in the map. Ages of the nodes estimated with BEAST dating analysis are indicated by the nodes, mean above in bold, 95% HPD interval in plain below.

**Figure 6:**
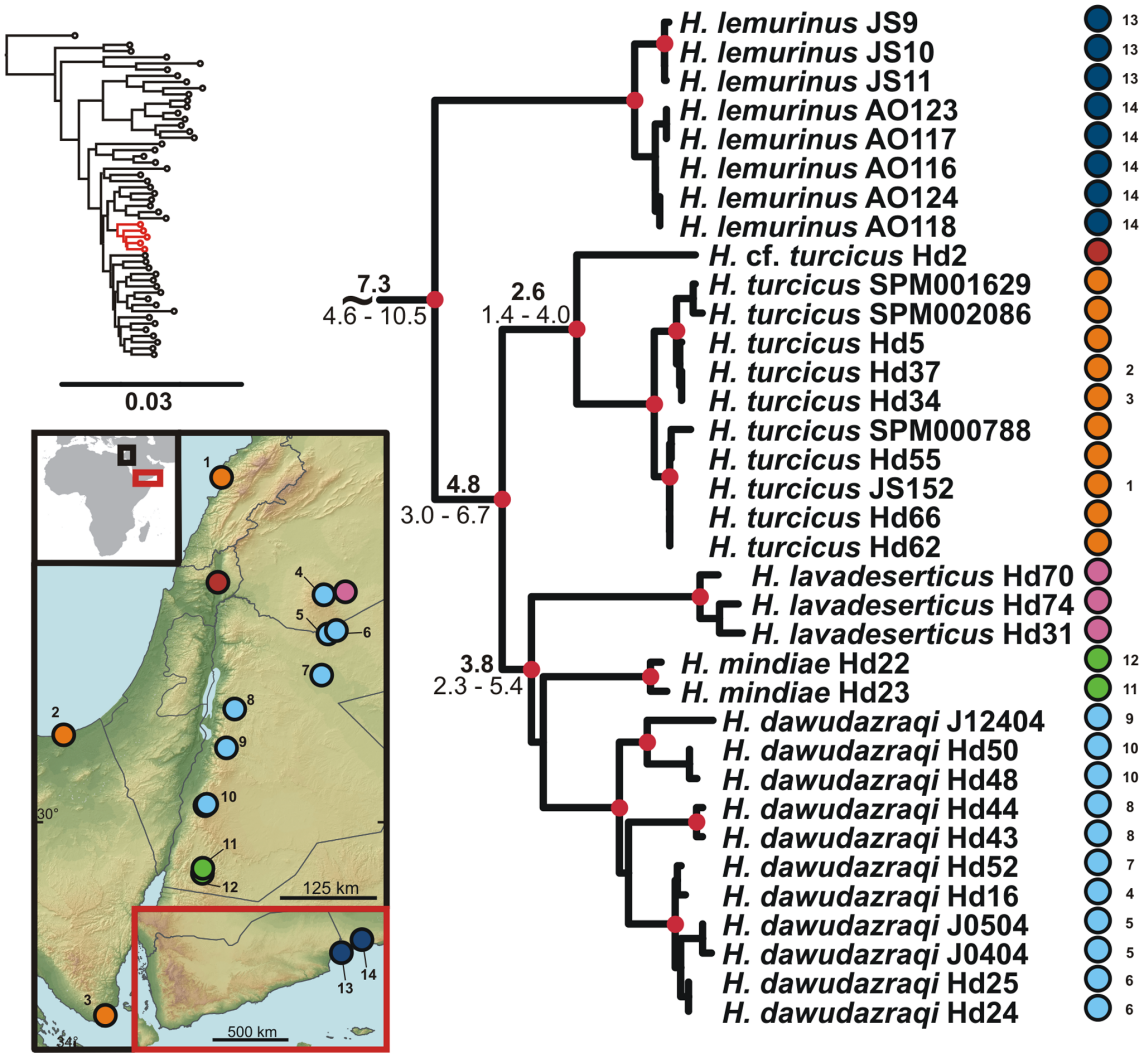
Detail of the phylogenetic tree of the Arid clade *Hemidactylus*: The Levant. Red dots in the nodes indicate ML bootstrap values ≥70 and BI pp≥0.95. Numbers after species names refer to sample codes; numbers on the right correspond with the localities numbers in the map. Ages of the nodes estimated with BEAST dating analysis are indicated by the nodes, mean above in bold, 95% HPD interval in plain below.

**Figure 7:**
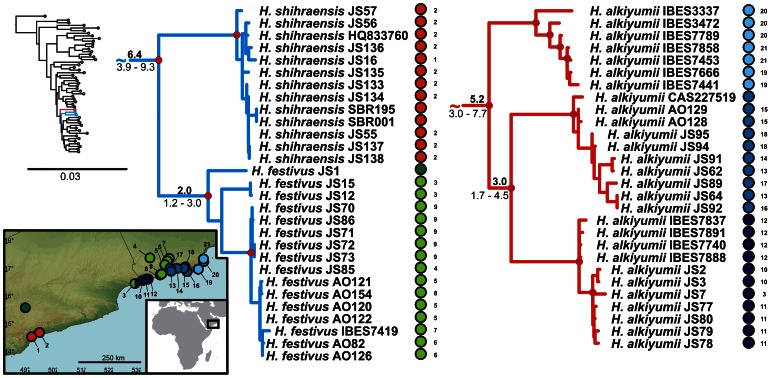
Detail of the phylogenetic tree of the Arid clade *Hemidactylus*: Hadhramaut and Dhofar. Red dots in the nodes indicate ML bootstrap values ≥70 and BI pp≥0.95. Numbers after species names refer to sample codes; numbers on the right correspond with the localities numbers in the map. Ages of the nodes estimated with BEAST dating analysis are indicated by the nodes, mean above in bold, 95% HPD interval in plain below.

**Figure 8:**
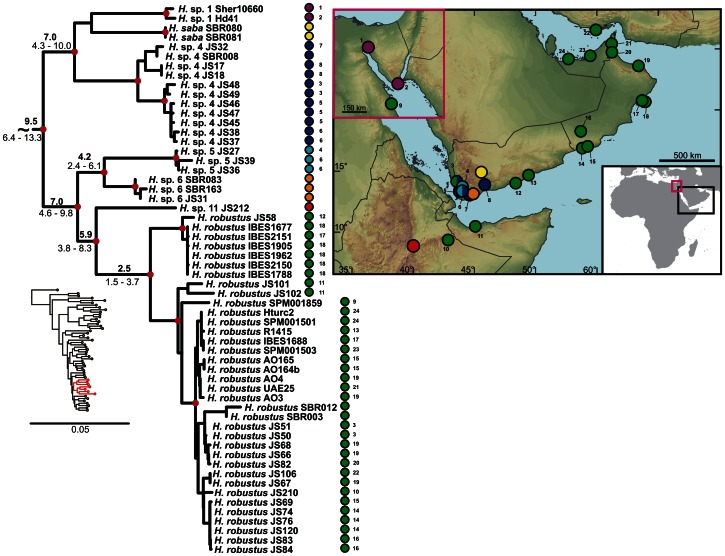
Detail of the phylogenetic tree of the Arid clade *Hemidactylus*: *H*. **** ***robustus*** **and related species.** **** Red dots in the nodes indicate ML bootstrap values ≥70 and BI pp≥0.95. Numbers after species names refer to sample codes; numbers on the right correspond with the localities numbers in the map. Ages of the nodes estimated with BEAST dating analysis are indicated by the nodes, mean above in bold, 95% HPD interval in plain below.

**Figure 9:**
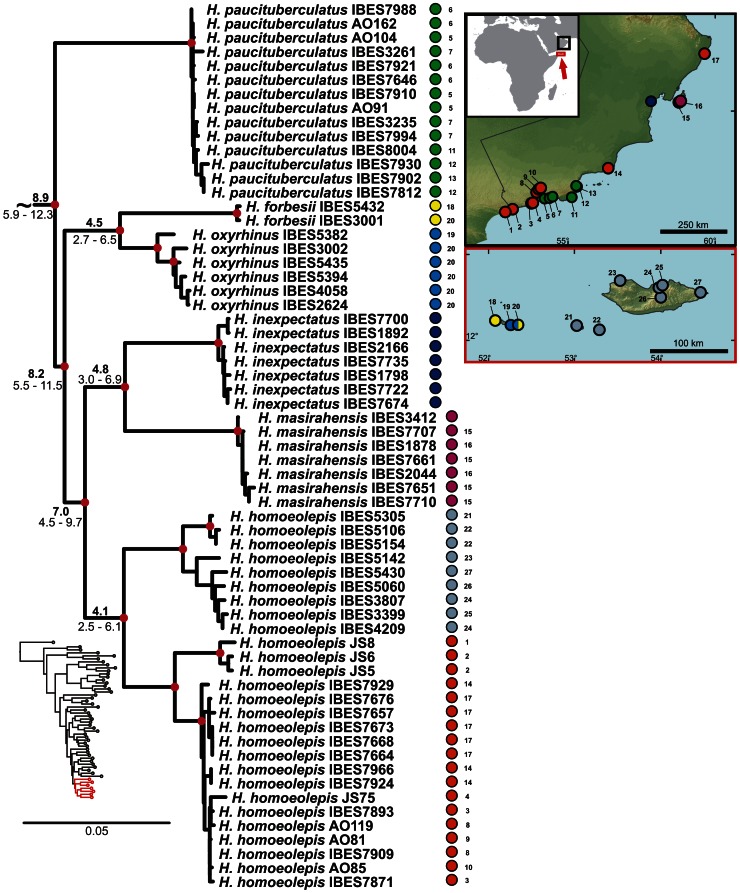
Detail of the phylogenetic tree of the Arid clade *Hemidactylus*: Oman and Socotra Archipelago. Red dots in the nodes indicate ML bootstrap values ≥70 and BI pp≥0.95. Numbers after species names refer to sample codes; numbers on the right correspond with the localities numbers in the map. Ages of the nodes estimated with BEAST dating analysis are indicated by the nodes, mean above in bold, 95% HPD interval in plain below.

The results of the phylogenetic analyses of the complete *Hemidactylus* dataset using ML and BI methods had the same topology at higher nodes and differed only slightly at the intraspecific level (Figs. S2, S3). As a result of that, only the ML tree with the bootstrap and pp support for both methods is presented with species clades drawn as collapsed (Fig. 1). All relevant information for the main groups of the Arid clade are depicted in details in Figs. 2, 3, 4, 5, 6, 7, 8, 9. Exactly the same subclades and species were also recovered from the analyses of the nDNA dataset only (Fig. S4). Variation in nuclear genes is an important indicator of species separation and an evidence of complete lineage sorting, suggesting existence of isolated species. The result of the estimates of the divergence dates has been incorporated in Figs. 2, 3, 4, 5, 6, 7, 8, 9 and the original result of the BEAST analysis is provided in Fig. S5.

*Hemidactylus ruspolii* and *H. angulatus* form a clade corresponding to the *H. angulatus* clade [6]. *Hemidactylus mabouia* and *H. platycephalus* cluster together as part of the African-Atlantic clade [6], [19], [23] together with *H. smithi*, incorporated into a phylogeny for the first time here, and thus confirmed to be a part of this clade. According to our analyses, all other *Hemidactylus* taxa, 29 Arabian species and 15 species from Northeast Africa analyzed in the present study, form a well supported monophyletic group (ML bootstrap = 100/Bayesian pp = 1) - the Arid clade. According to the phylogenetic hypotheses presented in Figs. 1, 2, 3, 4, 5, 6, 7, 8, 9, the Arid clade is formed by three phylogenetically and biogeographically clearly separated subclades. Basal dichotomy in the Arid clade separated 29.1 Ma (95% highest posterior density interval [HPD] 19.2–40.3; Figs 2, S5) a monophyletic group (100/1) of eleven strictly African species (*H. albopunctatus*, *H. citernii*, *H. foudaii*, *H. funaiolii*, *H. isolepis*, *H. modestus*, *H. ophiolepis*, *H. sinaitus*, *H. squamulatus*, *H.* sp. 9, *H.* sp. 10) from the rest. The second clade (99/1) that branches off consists of four Socotran species (*H. pumilio, H. inintellectus, H. dracaenacolus, H. granti*), which separated 20 Ma (HPD 13.3–27.9) and is sister to all the other, mostly mainland Arabian, species (Figs. 3, S5). Mutual relationships of subclades within the mainly Arabian radiation were not resolved with certainty in any of the analyses performed. Species in this Arabian radiation form four well supported individual clades which started to radiate 15 Ma (HPD 9.9–20.8) and formed: 1) a lineage of *H. persicus* samples from Iran; 2), a clade (100/1) consisting of *H. luqueorum* and *H. hajarensis*, which separated 13.1 Ma (HPD 8.6–18.3) from *H. persicus*, although the sister relationship between *H. persicus* and the latter two species does not have convincing support (38/0.87) (Fig. 4); 3) a clade (94/1) containing three African (*H. barodanus*, *H. granchii*, *H. macropholis*), four South Arabian (*H. jumailiae*, *H. lemurinus*, *H. y. yerburii*, *H. yerburii montanus*) and four Levantine (*H. dawudazraqi*, *H. lavadeserticus*, *H. mindiae*, *H. turcicus*) taxa, which diverged 11.3 Ma (HPD 7.5–15.6) (Figs. 5, 6, S5); and 4) a clade (99/1) that radiated 11.9 Ma (HPD 8–16.6, Fig. S5) containing eleven South Arabian species (*H. alkiyumii*, *H. festivus*, *H. homoeolepis*, *H. inexpectatus*, *H. masirahensis*, *H. paucituberculatus*, *H. shihraensis*, *H. saba*, *H*. sp. 4, *H.* sp. 5, *H*. sp. 6; species numbers 1–8 correspond to those in [23]), three Socotran species (*H. forbesii*, *H. homoeolepis*, *H. oxyrhinus*), the widespread *H. robustus* and two yet undescribed species, one from the Sinai (*H.* sp. 1) and another one from central Ethiopia (*H.* sp. 11) (Figs. 7, 8, 9).

In the reconstruction of the ancestral geographic distribution, the importance of changing the max areas in S-DIVA was explored (down to two, data not shown). We also tried to split the geographic origin assignments into more units (up to nine, data not shown) in order to obtain more detailed resolution. However, neither decreasing the number of max areas nor increasing the number of geographic units altered significantly the probabilities of ancestral ranges or changed the patterns of historical distribution of the ancestors. Therefore, the number of max areas was set to 4 and the area of interest was divided into the five regions described above (see Methods). The maximal S-DIVA value determining support for ancestral range reconstruction was 5309.02. The final results of the S-DIVA analysis are incorporated in the tree from Fig. 1.

## Discussion

The results of our analyses confirm the monophyly of the Arid clade of *Hemidactylus* as previously suggested [6]. Originally this clade consisted of only 13 species from Arabia, Socotra, East Africa and the Mediterranean. Additional 24 taxa were added to this clade in later studies [7], [19]–[21], [23], [26]. With the new species revealed in previous [19], [23] and this study, the Arid clade of *Hemidactylus* accounts for 35.4% out of a total of 130 recognized *Hemidactylus* species. Taking into account 16 species and subspecies from East Africa, some of which are likely to be a part of the Arid clade but are still pending to be included in any phylogenetic analysis (*H. arnoldi*, *H. barbierii*, *H. bavazzanoi*, *H. curlei*, *H. fragilis*, *H. jubensis*, *H. klauberi*, *H. laevis*, *H. laticaudatus*, *H. megalops*, *H. ophiolepoides*, *H. puccionii*, *H. somalicus*, *H. taylori*, *H. tropidolepis*, *H. yerburii pauciporosus*) and that there are some regions in Arabia like Saudi Arabia, which are still largely unexplored, we can conclude that the Arid clade can be regarded as the most speciose of all *Hemidactylus* clades [6].

### African – Arabian Vicariance and African Radiation

The basal dichotomy within the Arid clade separates a monophyletic group of eleven species (see Fig. 1) of strictly African origin. Because all the members of this African subclade inhabit Northeast Africa, their ancestor was presumably of the same origin (Fig. 2). Apart from the nine known species forming this subclade there are other two clearly separated lineages that, according to preliminary morphological analyses, deserve species status (work in progress). These two lineages are provisionally named *H.* sp. 9 (*Hemidactylus* sp. from central Ethiopia) and *H*. sp. 10 (*Hemidactylus* sp. from northern Kenya). According to the age estimates, this basal split took place 29.1 Ma (HPD 19.2–40.3 Ma, Figs. 2, S5). This date matches very well the geological estimates of the break-up of the Afro-Arabian continent and the consequent formation of the Red Sea and the Gulf of Aden [28], [29] and is supported by the same vicariant split from other studies [35], [36]. Therefore, the break-up of the African and Arabian tectonic plates seems to be responsible for the vicariant separation of the ancestors of these endemic African species from the rest of the Arid clade. The African subclade is formed by two well-supported and morphologically differentiated lineages: 1) species with distinctly enlarged dorsal tubercles and with ´bristlý appearance (*H. citernii*, *H. foudaii*, *H. sinaitus*, *H.* sp. 9) and 2) smooth-looking species without conical dorsal tubercles (*H. albopunctatus, H. funaiolii*, *H. isolepis*, *H. modestus*, *H. ophiolepis*, *H. squamulatus*, *H.* sp. 10). These two groups are distributed NW and SE of the Great Rift Valley (see Fig. 2), respectively with a minor overlap in the Ahmar Mountains in Ethiopia and Somalia and separated from each other 25.5 Ma (HPD 16.7–35.4). Of all taxa belonging to the African subclade, *H. sinaitus* from Sudan is particularly interesting from a taxonomic point of view. Until now, the only individuals of “*H. sinaitus*” that have been sequenced are from Yemen [20,21; unpublished sequences provided by U. Joger]. The type locality of *H. sinaitus* was reassessed from the original ´Mount Sinaí to the Sudanese shores of the Red Sea in the region of Suakin and Durrur [N of Suakin] [56], [57]. According to the phylogeny presented in Fig. 1, and after morphological examination of the specimens of “*H. sinaitus*” from Yemen included elsewhere [20], [21] (data not shown), we conclude that the name *Hemidactylus sinaitus* applies to the populations from NE Africa only, and that the “*H. sinaitus*” from Yemen represents a new species (provisionally referred here as *Hemidactylus* sp. 6). To reveal more details about this African *Hemidactylus* subclade and to have a better idea of their biogeography, systematics and evolution, a much larger sampling, including more species from these difficult to access regions, will be essential (Tab. S3; work in progress).

### Arabian – Socotran Vicariance

After the separation of the African subclade, a subsequent split within the Arid clade of *Hemidactylus* segregated the ancestor of a group of four Socotran species (*H. dracaenacolus*, *H. granti*, *H. inintellectus*, and *H. pumilio*; Figs. 1, 3, S5). Our inferred dates suggest that this Socotran subclade split approximately 16.9 Ma (HPD 11.0–23.8). As already suggested [26], this split most probably represents another vicariant event in the history of the genus *Hemidactylus*, produced by the initial continental break-up about 24 Ma and subsequent oceanic spreading occurring 17.5 Ma in the eastern part of the Gulf of Aden, which triggered the drifting of the Socotra Archipelago from the Arabian mainland [29]. These dates fit the HPD estimate of the segregation of this subclade. As shown in Fig. 3, the level of intraspecific variation of these Socotran species is very high. According to the results of the BPP (Bayesian Phylogenetics and Phylogeography [58]) species delimitation method applied by Gómez-Díaz et al. [26], the four endemic Socotran species in fact consist of 13 ´putative specieś, and suggest that the diversity of *Hemidactylus* on the relatively small island of Socotra is very high and has probably been favoured by ecological diversification and morphological separation of evolutionary independent lineages [26], [59], [60].

All the remaining species after the separation of the African and Socotran subclades form a well supported monophyletic group of mostly Arabian species. Eighteen out of 31 species within this subclade are distributed in South Arabia, four in Africa, five in the Levant and Sinai, three in the Socotra Archipelago, one in Iran, and one is widespread in coastal areas of all these regions (Tab. S3). The results of our phylogenetic and biogeographic analyses, together with the divergence time estimates, indicate that multiple independent dispersal events from Arabia have taken place in the history of *Hemidactylus* alongside the vicariant events described above.

### Dispersal to Iran

The oldest reported dispersal from Arabia occurred 13.1 Ma (HPD 8.6–18.3; Figs. 4, S5) when the ancestor of *H. persicus* colonized Iran. Since the closest relatives of *H. persicus* are found in northern Oman, the dispersal occurred most probably via the *Gomphotherium* land bridge [61] connecting the Arabian and Anatolian plates 18 Ma. After a temporary period of disconnection the bridge was continuously present since the mid-Miocene about 15 Ma ago and allowed faunal exchanges between Eurasia and Afro-Arabia [35], [36], [61], [62]. Alternatively, the colonization of Iran could take place across the Proto-Arabian Gulf after the *Gomphotherium* bridge disappeared. A recent colonization of Iran by *H. persicus* can be ruled out alone by the deep level of intraspecific differentiation within the Iranian populations, indicating its long presence in the area (Fig. 4). Animals morphologically assignable to this species also occur in NE Saudi Arabia, Iraq, Kuwait and Bahrain [3], however, samples from none of these countries were available for this study. Until some specimens of *H. persicus* from NE Arabia and also of another Iranian species, *H. romeshkanicus*, which resembles morphologically other *Hemidactylus* representatives from the Arid clade [63], are analyzed and included in the biogeographic context of the Persian Gulf surroundings, a closer insight into the zoogeographic history of *H. persicus* remains unclear.

### Dispersals to Africa

According to our findings, apart from the African subclade, a remnant from the vicariant split between Africa and Arabia (Fig. 2), Africa has been colonized at least twice independently from Arabia in the history of the *Hemidactylus* Arid clade (Fig. 1). One dispersal event, a jump with subsequent radiation in Africa, occurred 9.8 Ma (HPD 6.5–13.6; Figs. 1, 5, S5). At that time, Africa and Arabia were temporarily connected by a land bridge of halite deposits [29]. Therefore, the ancestor of the three species (*H. barodanus*, *H. granchii*, *H. macropholis*) representing the African branch may have used this bridge for crossing to Africa. Their sister group is restricted to the mountain areas and their foothills in SW Yemen (Fig. 5) which have undergone a continuous uplift since the Late Miocene up to the Holocene [27], producing an important vertical structuring of the region and probably triggering speciation in this relatively small area.

The younger from the two detected dispersals from Arabia to Africa has a divergence time estimate of 5.9 Ma (HPD 1.5–8.3; Figs. 1, 8, S5). HPD interval indicates that this dispersal event could be facilitated by the presence of a land bridge or, after re-opening of the Bab-el-Mandeb strait and final separation of Africa from Southwest Arabia 5.3 Ma [29], happed as an over-water transfer. As in the first dispersal to Africa, the closest relatives of the colonizer (*Hemidactylus* sp. 11) inhabit south-western Yemen. Apparently, the Red Sea after its opening in the mid-Oligocene to the Early Miocene (31–23 Ma) did not form an insurmountable barrier and enabled faunal exchanges, that may have been facilitated by the temporary land bridge connection (10–5.3 Ma), from one side to the other [32], [34], [35], [64].

It is worth noting that the successful transcontinental colonizations of *Hemidactylus* between Africa and Arabia took place only in one direction, from Arabia to Africa. Despite there is evidence that the opposite direction of the same route has been used multiple times after the Red Sea opening [32], [34], [35], [64] and that the African subclade also experienced an important radiation (see above), none of its members was able to penetrate to Arabia. The genus *Hemidactylus* thus represents a unique example of animals with the main direction of dispersals from Arabia towards Africa, unlike in most other reported cases where the direction was the opposite [32]–[36], [64].

### Dispersals to the Socotra Archipelago

Identically to the African pattern, the Socotra Archipelago experienced one vicariant event followed by two colonizations [26]. After its separation from the Arabian landmass with ancestors of the four species described above carried on, the islands were colonized by two subsequent independent overseas dispersals. First, the ancestor of *H. forbesii* and *H. oxyrhinus* colonized the Abd al Kuri Island (the westernmost islands of the Socotra Archipelago), approximately 8.2 Ma (HPD 5.5–11.5; Figs. 1, 9, S5). This colonization was followed by an *in situ* intraisland speciation 4.5 Ma (HPD 2.7–6.5) [26].

An additional colonization event took place 4.1 Ma (HPD 2.5–6.1; Figs. 9, S5), when the ancestor of *H. homoeolepis* dispersed from South Arabia to Socotra, Darsa and Samha Islands [26]. High genetic differences between Socotran and mainland populations of *H. homoeolepis*, together with a high level of morphological differentiation of some populations of mainland Arabia suggest that *H. homoeolepis* includes in fact several undescribed species (work in progress).

### Dispersals to the Levant and Sinai

In accordance with the pattern of two dispersals to each Africa and Socotra, there have been two independent dispersal events from South Arabia to the Levant and Sinai occurring approximately at the same time, ca 7 Ma. In one case, the ancestor of four Levantine species (*H. dawudazraqi*, *H. lavadeserticus*, *H. mindiae*, *H. turcicus*) dispersed from South Arabia. The cluster of these four species is sister to the geographically distant *H. lemurinus* from South Arabia. The isolation from *H. lemurinus* dates back to 7.3 Ma (HPD 4.6–10.5) and subsequent radiation in the Levant took place 4.8 Ma (HPD 3.0–6.7; Figs. 1, 6, S5). All these four species are endemic to the Levant and Sinai except *H. turcicus*, which, most probably, has spread across most Mediterranean coastal areas by human-mediated dispersal [6], [23]. Close phylogenetic relationship of south Arabian *H. lemurinus* with the Levantine taxa is even more enigmatic when morphology and ecology is taken into account. Whereas all the Levantine taxa possess distinct dorsal tubercles and are rock or ground dwelling [23], *H. lemurinus* is entirely smooth without any enlarged scales on the dorsum and restricted to large pale water-smoothed boulders [65], [66]. It seems to occupy the same ecological niche as sympatric *Ptyodactylus* to which it superficially resembles. For better understanding of the polarization and speed of morphological evolution within this subclade, more detailed research on the pace of phenotypic changes and evolution of habitat use is required.

*Hemidactylus* sp. 1, the second colonizer of the north Arabia, diverged from its sister species 7.0 Ma (HPD 4.3–10.0) and subsequently colonized Sinai. Its sister species, *H. saba*, and *H.* sp. 4, are distributed in the mountains of western Yemen [23] (Fig. 8). Whether its occurrence in coastal Sinai is caused artificially by human-mediated (probably ship) transport or if its range stretches along the Hijaz and Asir Mountains in Saudi Arabia, an important colonization route [39], [67], [68], remains unknown and requires additional sampling from the eastern Red Sea coast.

### Human-mediated Dispersal of *H. robustus*

Although there is a certain genetic structure within *H. robustus* with a deep historical pre-Pleistocene origin of radiation (2.5 Ma; HPD 1.5–3.7; Fig. 8), it is not reflected in the geographical structuring of its populations. *Hemidactylus robustus* has been distributed all over the area of our study recently, most probably by human-mediated dispersal [7], [57], [69] similarly to *H. flaviviridis* (this study, data not shown) and *Chalcides ocellatus* [70], [71]. Even though some geographical pattern of *H. robustus* populations might have evolved historically, it was probably blurred by the recent dispersal of individual lineages. It is interesting to notice that, even though we hypothesize that such mixture of populations has been a recurrent phenomenon in recent times, the original genetic pattern has not disappeared entirely yet.

### Concluding Remarks

**Figure 10:**
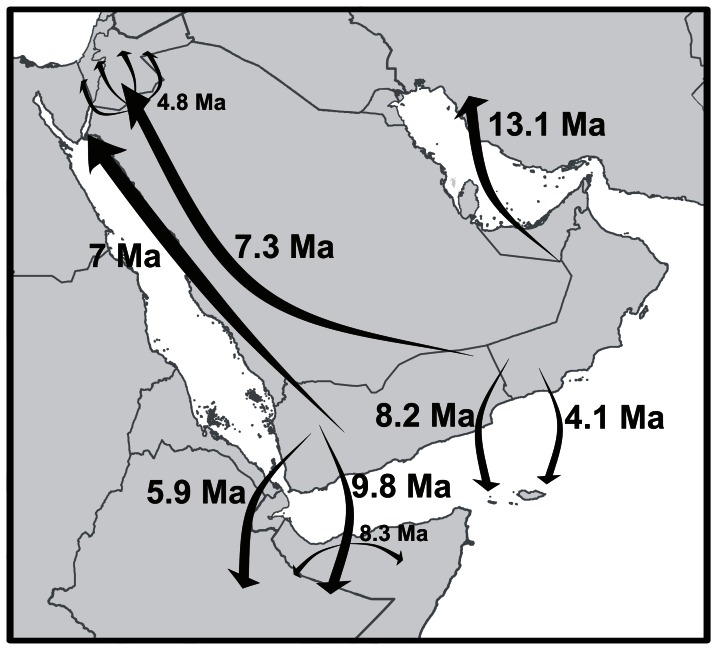
Summary of historical dispersal events of *Hemidactylus* geckos from Arabia. Dates by arrows indicate mean time estimates of the events. *In situ* radiation of some lineages following their dispersal is indicated as a radiation of arrows.

As is obvious from the presented phylogeny, divergence time estimates and historical biogeographic reconstructions, evolutionary history of the genus *Hemidactylus* in Arabia and its surroundings has a complex pattern of several vicariant events connected to major continental break-ups in the geological history of the region followed by multiple subsequent dispersal events from Arabia to other surrounding regions (Fig. 10). It thus forms a unique laboratory of evolutionary and biogeographic processes where the geological history of the area has played a crucial role in forming the phylogenetic pattern of *Hemidactylus* found today and contributed significantly to local diversity of the genus. Discovered cryptic diversity of *Hemidactylus* in the mountains of Yemen and Ethiopia emphasizes the importance of these highland areas as a part of the Eastern Afromontane biodiversity hotspot [72], [73]. Comparing overall reptile species richness in South Arabia and the Horn of Africa with how little is known about it we can assume that future studies may reveal more cryptic species (see also [74]) in various reptile groups with unforeseen phylogenetic and biogeographic relationships.

## Supporting Information

Figure S1 **Physical map of the region of the study with geographic names of important features and countries that appear in the text.** Country names are in italics.
(TIF)

Figure S2 **Original ML phylogenetic tree with all individuals analyzed.** ML bootstrap support values ≥70 shown.
(TIF)

Figure S3 **Original BI phylogenetic tree with all individuals analyzed.** BI posterior probabilities ≥0.95 shown.
(TIF)

Figure S4 **ML tree as a result of an analysis of four nDNA genes.** ML bootstrap support/BI pp drawn by the nodes. Only bootstrap values ≥70 (ML) and BI pp≥0.95 shown.
(TIF)

Figure S5 **Chronogram showing the results from BEAST.** Mean node estimates in bold, 95% HPD intervals in brackets and as the blue node bar.
(TIF)

Table S1 **Complete list of material used for this study.** Information on the specimens included in the phylogenetic analyses are listed in alphabetical order, with the corresponding GenBank accession numbers. Individuals with the specimen code highlighted with a hatch symbol (#) were included in the BEAST and S-DIVA analyses (see Methods).
(PDF)

Table S2 **Molecular markers, primers, primer sequences, amplification conditions and original primer sources used in this study.**
(PDF)

Table S3 **List of all** ***Hemidactylus*** **species from Arabia, the Horn of Africa, the Levant and Iran.** Black dots indicate known distribution records for each country, the rightmost column shows species included in this study.
(PDF)
