# Supplementary figures and images for: Out of Arabia: A Complex Biogeographic History of Multiple Vicariance and Dispersal Events in the Gecko Genus Hemidactylus (Reptilia: Gekkonidae)
Source: PLoS One. 2013 May 27;8(5):e64018. doi: 10.1371/journal.pone.0064018 (PMC3664631; doi:10.1371/journal.pone.0064018)

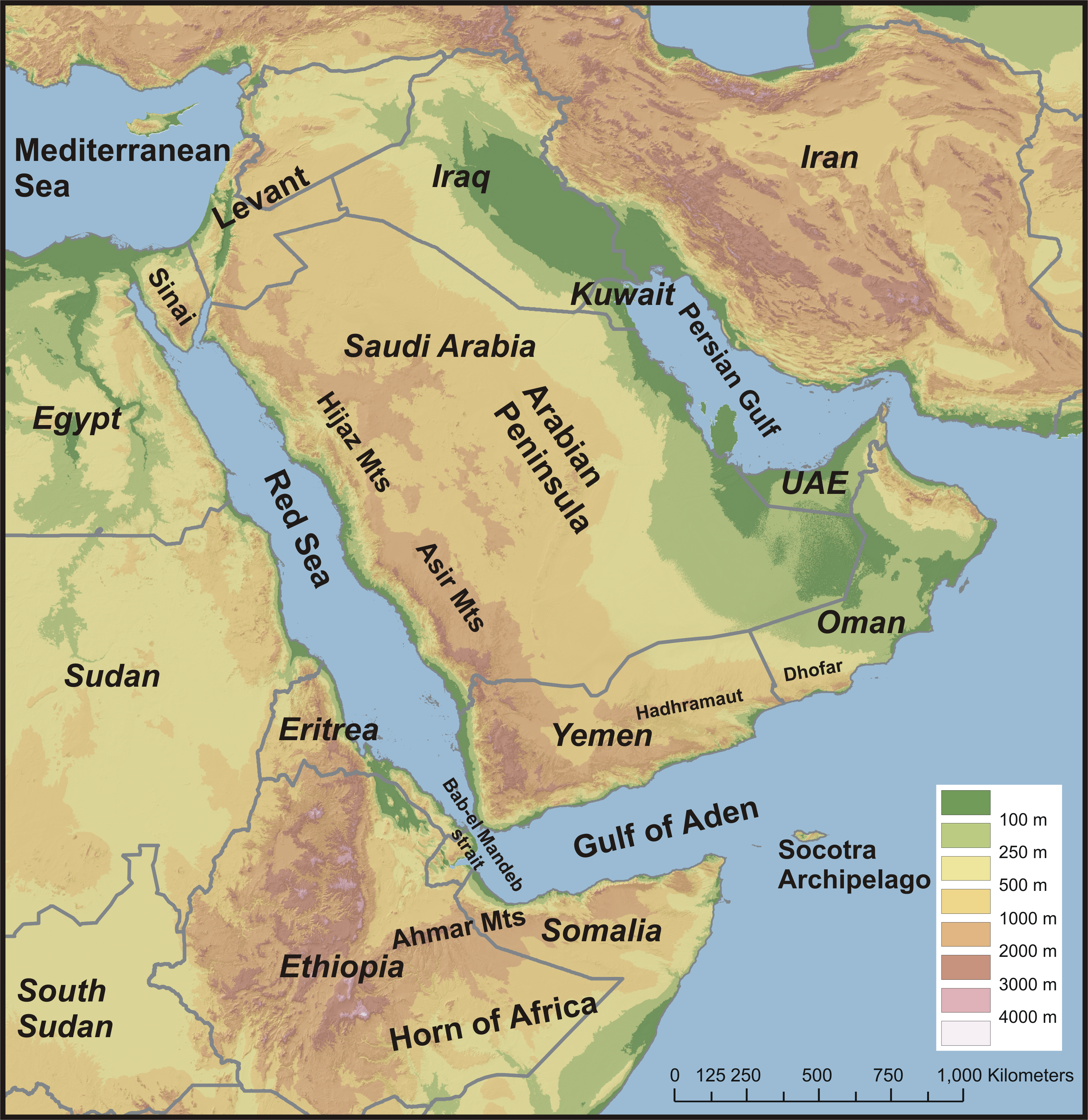

Supplement: Figure S1 — Physical map of the region of the study with geographic names of important features and countries that appear in the text. Country names are in italics. (TIF) [file pone.0064018.s001.tif]

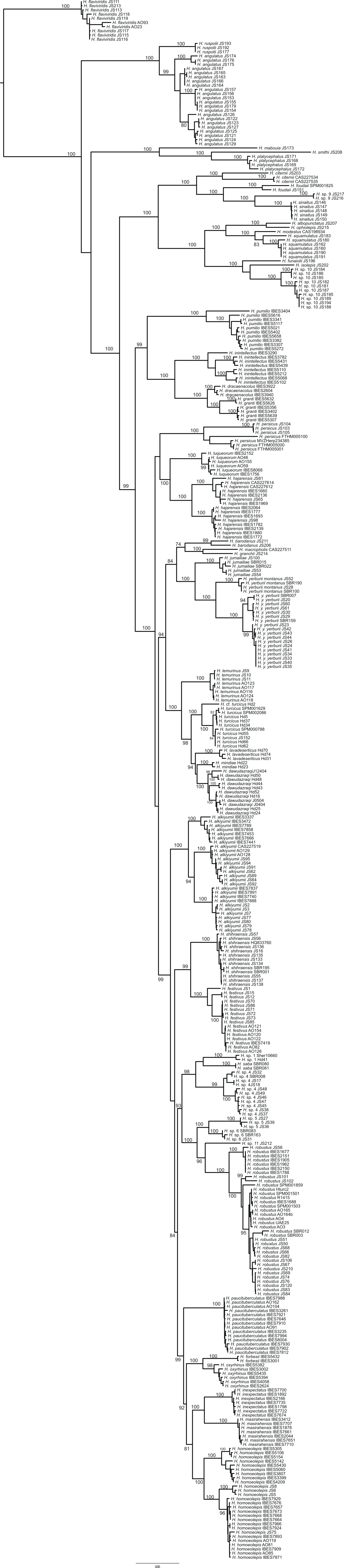

Supplement: Figure S2 — Original ML phylogenetic tree with all individuals analyzed. ML bootstrap support values ≥70 shown. (TIF) [file pone.0064018.s002.tif]

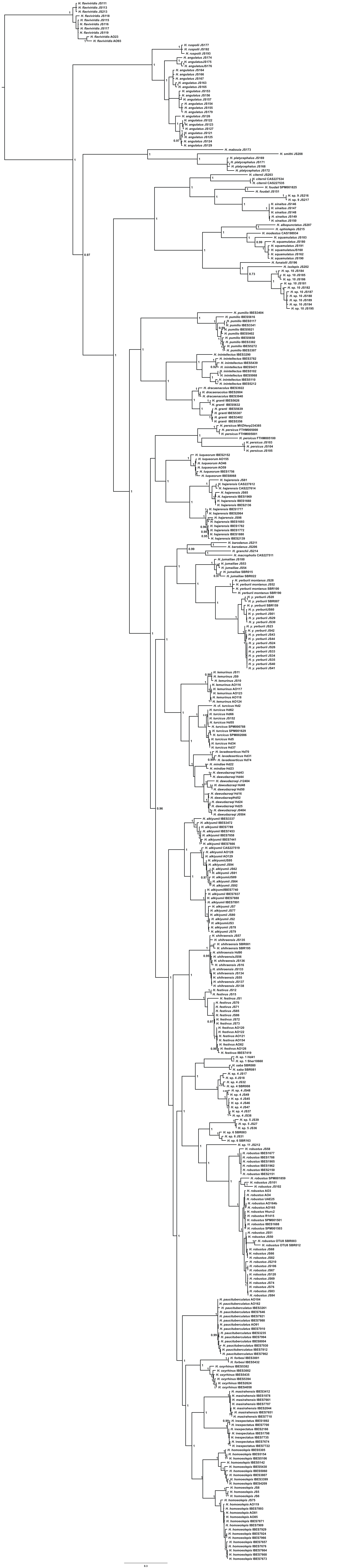

Supplement: Figure S3 — Original BI phylogenetic tree with all individuals analyzed. BI posterior probabilities ≥0.95 shown. (TIF) [file pone.0064018.s003.tif]

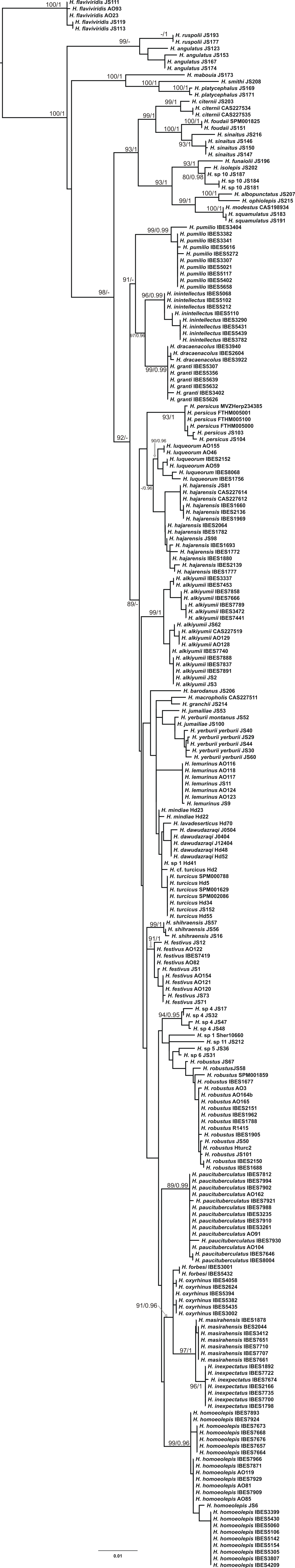

Supplement: Figure S4 — ML tree as a result of an analysis of four nDNA genes. ML bootstrap support/BI pp drawn by the nodes. Only bootstrap values ≥70 (ML) and BI pp≥0.95 shown. (TIF) [file pone.0064018.s004.tif]

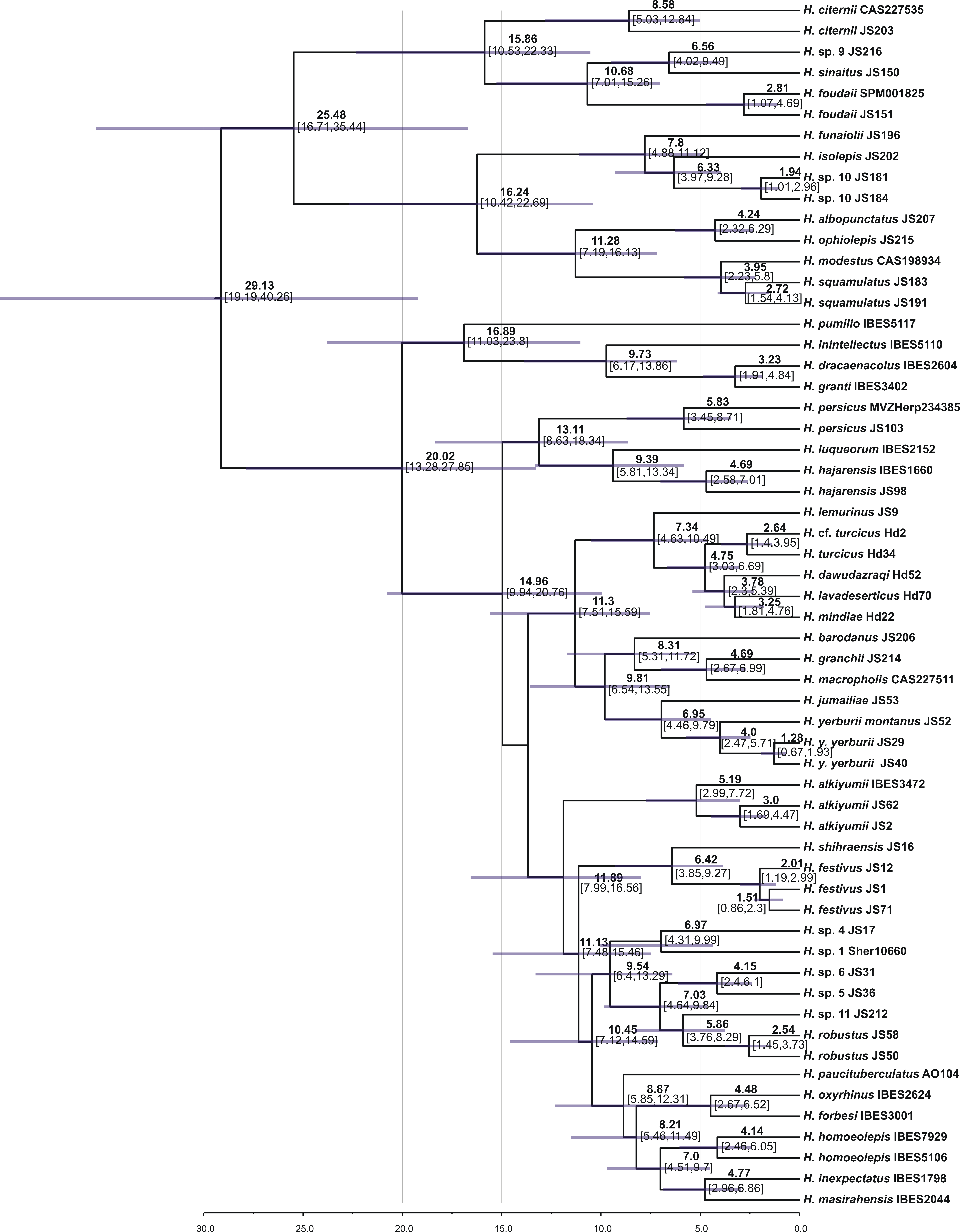

Supplement: Figure S5 — Chronogram showing the results from BEAST. Mean node estimates in bold, 95% HPD intervals in brackets and as the blue node bar. (TIF) [file pone.0064018.s005.tif]
