# Supplementary material for: Out of Arabia: A Complex Biogeographic History of Multiple Vicariance and Dispersal Events in the Gecko Genus Hemidactylus (Reptilia: Gekkonidae)
Source: PLoS One. 2013 May 27;8(5):e64018. doi: 10.1371/journal.pone.0064018 (PMC3664631; doi:10.1371/journal.pone.0064018)
Supplement: Table S1 — Complete list of material used for this study. Information on the specimens included in the phylogenetic analyses are listed in alphabetical order, with the corresponding GenBank accession numbers. Individuals with the specimen code highlighted with a hatch symbol (#) were included in the BEAST and S-DIVA analyses (see Methods). (PDF) [file pone.0064018.s006.pdf]

Table S1. Complete list of material used for this study. Information on the specimens included in the phylogenetic analyses listed in alphabetical order, with the corresponding GenBank accession numbers. Individuals with the specimen code highlighted with a hatch symbol (#) were included in the BEAST and S-DIVA analyses (see Materials and methods).

SPECIES	CODE	VOUCHER	COUNTRY	LOCALITY	*12S*	*cytb*	*nd4 + tRNAs*	*cmos*	*mc1r*	*rag2*	*rag1*
H. albopunctatus	JS207#		Ethiopia	42 km SE of Jijiga	KC818657	KC818794	-	KC818745	KC818901	KC819017	KC818952
H. alkiyumii	S3337		Oman	Wadi Hasik	JQ957039	JQ957170	-	JQ957123	JQ957239	JQ957401	-
H. alkiyumii	S3472#		Oman	Wadi Hasik	JQ957040	JQ957171	JQ957310	JQ957123	JQ957240	JQ957403	-
H. alkiyumii	S7789		Oman	7.5 km S Hasik	JQ957043	JQ957175	JQ957315	JQ957123	JQ957240	JQ957404	-
H. alkiyumii	S7441	IBES7441	Oman	3.5 km NE Sadah	JQ957041	JQ957172	JQ957311	JQ957123	JQ957240	JQ957404	-
H. alkiyumii	S7666	IBES7666	Oman	3.5 km NE Sadah	JQ957041	JQ957172	JQ957313	JQ957123	JQ957242	JQ957401	-
H. alkiyumii	S7858	IBES7858	Oman	3 km NW of Hasik	JQ957040	JQ957177	JQ957317	JQ957123	JQ957245	JQ957404	-
H. alkiyumii	S7453	IBES7453	Oman	3 km NW of Hasik	JQ957040	JQ957173	JQ957312	JQ957123	JQ957241	JQ957405	-
H. alkiyumii	CAS227519	CAS227519	"Somalia"	"11 km SE of Bosasso" (wrong locality)	DQ120343	DQ120172	JQ957309	-	JQ957238	JQ957402	-
H. alkiyumii	AO129	BM2005.1662	Oman	Tawi Atair	JQ957038	JQ957169	-	JQ957122	JQ957237	JQ957401	-
H. alkiyumii	AO128	BM2005.1663	Oman	Tawi Atair	JQ957038	JQ957168	-	JQ957121	JQ957236	JQ957401	-
H. alkiyumii	S7837	IBES7837	Oman	Dalkut	JQ957044	JQ957176	JQ957316	JQ957123	JQ957244	JQ957401	-
H. alkiyumii	S7740	IBES7740	Oman	Dalkut	JQ957042	JQ957174	JQ957314	JQ957123	JQ957243	JQ957401	-
H. alkiyumii	S7888	IBES7888	Oman	Dalkut	JQ957042	JQ957178	JQ957318	JQ957123	JQ957246	JQ957401	-
H. alkiyumii	S7891	IBES7891	Oman	Dalkut	JQ957045	-	JQ957319	JQ957123	JQ957246	JQ957401	-
H. alkiyumii	JS2#	NMP6V 74799/1	Yemen	3 km E Hauf	JQ957090	KC818795	-	JQ957123	JQ957244	JQ957401	KC818953
H. alkiyumii	JS3	NMP6V 74799/9	Yemen	3 km E Hauf	JQ957091	KC818795	-	JQ957123	JQ957246	JQ957401	KC818954
H. alkiyumii	JS7	NMP6V 74800	Yemen	Damqawt	JQ957094	KC818796	-	-	-	-	-
H. alkiyumii	JS62#	NMP6V 74839/1	Oman	Salalah city	JQ957092	KC818802	-	KC818746	KC818902	JQ957401	KC818955
H. alkiyumii	JS64	NMP6V 74839/3	Oman	Salalah city	JQ957093	KC818803	-	-	-	-	-
H. alkiyumii	JS77	NMP6V 74838/1	Oman	2 km NW of Dalkut	JQ957090	KC818797	-	-	-	-	-
H. alkiyumii	JS78	NMP6V 74838/2	Oman	2 km NW of Dalkut	JQ957090	KC818798	-	-	-	-	-
H. alkiyumii	JS79	NMP6V 74838/3	Oman	2 km NW of Dalkut	JQ957090	KC818799	-	-	-	-	-
H. alkiyumii	JS80	NMP6V 74838/4	Oman	2 km NW of Dalkut	JQ957090	KC818800	-	-	-	-	-
H. alkiyumii	JS89	NMP6V 74840/3	Oman	8 km N. Salalah airport	JQ957092	KC818801	-	-	-	-	-
H. alkiyumii	JS91	NMP6V 74842/2	Oman	3 km E. Ain Hamran	JQ957092	KC818802	-	-	-	-	-
H. alkiyumii	JS92	NMP6V 74843	Oman	Mirbat	JQ957093	KC818803	-	-	-	-	-
H. alkiyumii	JS94	NMP6V 74844/1	Oman	Taiq Cave	JQ957092	KC818804	-	-	-	-	-
H. alkiyumii	JS95	NMP6V 74844/2	Oman	Taiq Cave	JQ957095	KC818805	-	-	-	-	-
H. angulatus	JS121	NMP6V 74847	Ethiopia	Agere Maryam	KC818658	KC818806	-	-	-	-	-
H. angulatus	JS122	NMP6V 74845/1	Ethiopia	Arba Minch	KC818659	KC818807	-	-	-	-	-
H. angulatus	JS123	NMP6V 74845/2	Ethiopia	Arba Minch	KC818659	KC818807	-	KC818747	KC818903	KC819018	KC818956
H. angulatus	JS124	NMP6V 74846	Ethiopia	Konso	KC818658	KC818808	-	-	-	-	-
H. angulatus	JS125	NMP6V 74848	Ethiopia	Yabello	KC818658	KC818809	-	-	-	-	-
H. angulatus	JS126	NMP6V 74852	Ethiopia	Jinka	KC818660	KC818810	-	-	-	-	-
H. angulatus	JS127	NMP6V 74853	Ethiopia	Arba Minch	KC818659	KC818811	-	-	-	-	-
H. angulatus	JS129	NMP6V 74851/2	Ethiopia	Hammar	KC818658	KC818812	-	-	-	-	-
H. angulatus	JS153	NMP6V 74814/1	Kenya	South Horr	KC818661	-	-	KC818748	KC818904	KC819019	-
H. angulatus	JS154	NMP6V 74814/2	Kenya	South Horr	KC818662	-	-	-	-	-	-
H. angulatus	JS155	NMP6V 74814/3	Kenya	South Horr	KC818662	-	-	-	-	-	-
H. angulatus	JS156	NMP6V 74814/4	Kenya	South Horr	KC818661	-	-	-	-	-	-
H. angulatus	JS157	NMP6V 74814/5	Kenya	South Horr	KC818663	-	-	-	-	-	-
H. angulatus	JS163	NMP6V 74813/1	Kenya	Nginyang	KC818664	-	-	-	-	-	-
H. angulatus	JS164	NMP6V 74813/2	Kenya	Nginyang	KC818665	-	-	-	-	-	-
H. angulatus	JS165	NMP6V 74813/3	Kenya	Nginyang	KC818664	-	-	-	-	-	-
H. angulatus	JS166	NMP6V 74813/4	Kenya	Nginyang	KC818665	-	-	-	-	-	-
H. angulatus	JS167	NMP6V 74813/5	Kenya	Nginyang	KC818665	-	-	KC818749	-	-	-
H. angulatus	JS174	NMP6V 74815/3	Uganda	Ubbi, Otzi Forest	KC818666	KC818813	-	KC818749	KC818905	KC819020	-
H. angulatus	JS175	NMP6V 74815/2	Kenya	Ubbi, Otzi Forest	KC818667	-	-	-	-	-	-
H. angulatus	JS176	NMP6V 74815/1	Kenya	Ubbi, Otzi Forest	KC818667	-	-	-	-	-	-
H. angulatus	JS179	NMP6V 74849/2	Kenya	South Horr	KC818662	-	-	-	-	-	-
H. barodanus	JS206#		Ethiopia	15 km NE of Dire Dawa	KC818668	-	-	KC818750	KC818906	KC819021	KC818957
H. barodanus	JS211		Somalia	Laas Geel	KC818669	KC818814	-	-	-	-	-
H. citernii	CAS227534	CAS227534	Somalia	Bari Region	DQ120383	DQ120212	JQ957320	JQ957124	JQ957247	JQ957406	-
H. citernii	CAS227535#	CAS227535	Somalia	Bari Region	DQ120384	DQ120213	JQ957321	JQ957124	JQ957248	JQ957407	-
H. citernii	JS203#		Somalia	Laas Geel	KC818670	KC818815	-	KC818751	KC818907	KC819022	KC818958
H. dawudazraqi	J12404		Jordan	Wadi al Burbeyath	DQ120335	DQ120164	JQ957397	JQ957161	JQ957299	JQ957442	-
H. dawudazraqi	J0504		Jordan	Dair al Khaf	DQ120336	DQ120165	JQ957396	JQ957161	JQ957300	JQ957441	-
H. dawudazraqi	J0404		Jordan	Dair al Khaf	JQ957082	JQ957230	JQ957395	JQ957161	JQ957299	JQ957440	-
H. dawudazraqi	Hd16	NMP6V 70457	Syria	Rashiedeh	KC818671	HQ833749	-	-	-	-	-
H. dawudazraqi	Hd24	NMP6V 72740/1	Jordan	Jawa	KC818672	HQ833750	-	-	-	-	-
H. dawudazraqi	Hd25	NMP6V 72740/2	Jordan	Jawa	KC818672	HQ833751	-	-	-	-	-
H. dawudazraqi	Hd43	NMP6V 74135/6	Jordan	Wadi Mujib	KC818673	HQ833754	-	-	-	-	-
H. dawudazraqi	Hd44	NMP6V 74135/7	Jordan	Wadi Mujib	KC818674	HQ833755	-	-	-	-	-
H. dawudazraqi	Hd48	NMP6V 74136/7	Jordan	Little Petra	KC818675	HQ833757	-	JQ957161	KC818908	JQ957423	KC818959
H. dawudazraqi	Hd50	NMP6V 74137	Jordan	Petra	KC818675	HQ833758	-	-	-	-	-
H. dawudazraqi	Hd52#	NMP6V 74134/1	Jordan	Azraq	KC818671	HQ833753	-	JQ957161	JQ957299	JQ957423	KC818960
H. dracaenacolus	IBES3922		Yemen	Wadi Zeriq, Socotra Island	JQ982783	JQ982890	JQ982704	KC818753	JQ982642	KC819023	-
H. dracaenacolus	IBES3940		Yemen	Bivio Diksam, Socotra Island	JQ982784	JQ982891	JQ982705	KC818752	JQ982644	KC819023	-
H. dracaenacolus	IBES2604#		Yemen	Tahr Diksam, Socotra Island	JQ982781	JQ982889	JQ982702	KC818752	JQ982643	KC819023	-
H. festivus	S7419	IBES7419	Oman	20 km S of Tumrait	JQ957047	JQ957181	JQ957324	JQ957125	JQ957252	JQ957410	-
H. festivus	AO126		Oman	3 km SE of Haluf	JQ957047	JQ957181	-	-	-	-	-
H. festivus	AO82		Oman	3 km SE of Haluf	JQ957047	JQ957181	JQ957323	JQ957125	JQ957251	JQ957410	-
H. festivus	AO122		Oman	Wadi Ayoun	JQ957047	JQ957179	JQ957322	JQ957125	-	JQ957409	-
H. festivus	AO120		Oman	Wadi Ayoun	JQ957047	JQ957179	JQ957322	JQ957125	JQ957249	JQ957408	-
H. festivus	AO154		Oman	Close to Mughsayl	JQ957047	JQ957182	JQ957322	JQ957125	JQ957250	JQ957409	-
H. festivus	AO121		Oman	Wadi Ayoun	JQ957046	JQ957180	JQ957322	JQ957125	JQ957250	JQ957409	-
H. festivus	JS1#	NMP6V 74812	Yemen	Wadi Hadramauth	JQ957096	KC818816	-	JQ957125	KC818909	JQ957409	KC818961
H. festivus	JS12#		Yemen	Damqawt	JQ957097	HQ833761	-	JQ957125	KC818910	KC819024	KC818962
H. festivus	JS15	NMP6V 74170	Yemen	Damqawt	JQ957097	HQ833761	-	-	-	-	-
H. festivus	JS70	NMP6V 74854/1	Oman	Mughsayl	JQ957098	KC818817	-	-	-	-	-
H. festivus	JS71#	NMP6V 74854/2	Oman	Mughsayl	JQ957098	KC818817	-	JQ957125	JQ957250	JQ957408	KC818963
H. festivus	JS72	NMP6V 74854/3	Oman	Mughsayl	JQ957098	KC818818	-	-	-	-	-
H. festivus	JS73	NMP6V 74854/4	Oman	Mughsayl	JQ957098	KC818819	-	JQ957125	JQ957250	KC819025	KC818964
H. festivus	JS85		Oman	Mudayy	JQ957098	KC818820	-	-	-	-	-
H. festivus	JS86	NMP6V 74855	Oman	Mughsayl	JQ957098	KC818821	-	-	-	-	-
H. flaviviridis	AO23		Oman	E. of Nizwa	JQ957048	JQ957183	JQ957325	JQ957126	JQ957253	JQ957411	-
H. flaviviridis	AO93		Oman	East Khor	JQ957049	JQ957184	JQ957326	JQ957126	JQ957253	JQ957412	-
H. flaviviridis	JS111		Pakistan	Okara	KC818676	KC818822	-	JQ957126	JQ957253	KC819026	KC818965
H. flaviviridis	JS113		India	Haridwar	KC818676	KC818823	-	JQ957126	JQ957253	KC819027	KC818966
H. flaviviridis	JS115		Yemen	14 km N of Mocha	JQ957119	KC818824	-	-	-	-	-
H. flaviviridis	JS116		Yemen	Zabid	JQ957119	JQ957183	-	-	-	-	-
H. flaviviridis	JS117		Oman	Nakhl	JQ957119	KC818825	-	-	-	-	-
H. flaviviridis	JS118		Oman	Salalah	JQ957120	JQ957184	-	-	-	-	-

SPECIES	CODE	VOUCHER	COUNTRY	LOCALITY	*12S*	*cytb*	ND4 + tRNAs	*cmos*	*mc1r*	*rag2*	*rag1*
H. flaviviridis	JS119		Oman	Jalan Bani Bu Hasan	JQ957119	JQ957183	-	KC818754	KC818911	KC819028	KC818967
H. flaviviridis	JS213		Ethiopia	Metahara	KC818676	KC818826	-	-	-	-	-
H. forbesii	IBE3001[#]		Yemen	Bir Al Aguz, Abd al Kuri Island	JQ982785	JQ982893	JQ982707	KC818755	JQ982645	JQ957425	-
H. forbesii	IBE5432		Yemen	Al Alyah, Abd al Kuri Island	JQ982786	JQ982894	JQ982711	KC818756	JQ982647	JQ957425	-
H. foudaii	SPM001825[#]		Egypt	Jebel Elba	DQ120385	DQ120214	JQ957327	JQ957127	-	JQ957413	-
H. foudaii	JS151[#]	NMP6V 74808	Sudan	15 km SE of Atbara	KC818677	-	-	KC818757	KC818912	KC819029	KC818968
H. funaiolii	JS196[#]		Kenya	Hurri Hills	KC818678	KC818827	-	KC818758	KC818913	KC819030	KC818969
H. granchii	JS214[#]		Somalia	40 km NE of Hargeisa	KC818679	KC818828	-	JQ957138	KC818914	KC819031	KC818970
H. granti	IBES3402[#]		Yemen		JQ982787	JQ982897	JQ982712	KC818752	JQ982651	KC819023	-
H. granti	IBES5356		Yemen		JQ982789	JQ982896	JQ982712	KC818752	JQ982652	KC819032	-
H. granti	IBES5307		Yemen	Adho Dimellus, Socotra Island	JQ982788	JQ982902	JQ982716	KC818759	JQ982652	KC819023	-
H. granti	IBES5626		Yemen	Scant, Socotra Island	JQ982790	JQ982903	JQ982718	KC818752	JQ982655	KC819023	-
H. granti	IBES5632		Yemen	Scant, Socotra Island	JQ982790	JQ982903	JQ982719	KC818760	JQ982655	KC819023	-
H. granti	IBES5639		Yemen	Scant, Socotra Island	JQ982787	JQ982902	JQ982720	KC818752	JQ982652	KC819033	-
H. hajarensis	CAS227612	CAS227612	Oman	4.5 km. N of Tanuf	DQ120337	DQ120166	JQ957328	JQ957128	JQ957254	JQ957414	-
H. hajarensis	CAS227614	CAS227614	Oman	4.5 km N. of Tanuf	DQ120338	DQ120167	JQ957329	JQ957128	JQ957255	JQ957415	-
H. hajarensis	S1969	BM2008.701	Oman	Wadi Mayh	JQ957055	JQ957189	JQ957335	JQ957128	JQ957260	JQ957415	-
H. hajarensis	S2136	BM2008.702	Oman	Wadi Mayh	JQ957050	JQ957185	JQ957337	-	JQ957260	JQ957415	-
H. hajarensis	S1660[#]	BM2008.703	Oman	Jebel Abu Daud	JQ957050	JQ957185	JQ957330	JQ957128	JQ957256	JQ957415	-
H. hajarensis	S2064	BM2008.709	Oman	Wadi Hebaheba, Jebel Qawan	JQ957053	JQ957188	JQ957336	JQ957128	-	-	-
H. hajarensis	S1777	S1777	Oman	Wadi Hebaheba, Jebel Qawan	JQ957053	JQ957188	JQ957333	JQ957130	JQ957258	JQ957418	-
H. hajarensis	S1772	BM2008.706	Oman	Wadi Hebaheba, Jebel Qawan	JQ957052	JQ957187	JQ957332	JQ957129	-	JQ957417	-
H. hajarensis	S1880	BM2008.707	Oman	Wadi Hebaheba, Jebel Qawan	JQ957052	JQ957187	JQ957332	JQ957128	JQ957259	JQ957420	-
H. hajarensis	S2139	BM2008.708	Oman	Wadi Hebaheba, Jebel Qawan	JQ957052	JQ957187	JQ957338	JQ957128	JQ957261	JQ957421	-
H. hajarensis	S1693	BM2008.704	Oman	Wadi Tiwi	JQ957051	JQ957186	JQ957331	JQ957128	JQ957257	JQ957416	-
H. hajarensis	S1782	BM2008.705	Oman	Wadi Tiwi	JQ957054	JQ957187	JQ957334	JQ957128	-	JQ957419	-
H. hajarensis	JS65	NMP6V 74862	Oman	Wadi N of Qurayyat	JQ957105	KC818829	-	-	-	-	-
H. hajarensis	JS81	NMP6V 74861	Oman	Wadi Bani Awf	JQ957099	KC818830	-	JQ957128	-	JQ957415	KC818971
H. hajarensis	JS98[#]	NMP6V 74860/1	Oman	Muqal	JQ957101	KC818831	-	JQ957128	KC818915	JQ957419	KC818972
H. homoeolepis	S4209		Yemen	Wadi Ayhaft, Socotra Island	JQ957059	JQ957194	JQ957342	JQ957132	JQ957264	JQ957422	-
H. homoeolepis	S3399		Yemen	Hadibo, Socotra Island	JQ957059	JQ957193	JQ957341	JQ957132	JQ957263	JQ957422	-
H. homoeolepis	S7929[#]	IBES7929	Oman	14.5 km NE of Sharbthat	JQ957057	JQ957198	JQ957349	JQ957131	JQ957262	JQ957422	-
H. homoeolepis	S7676	IBES7676	Oman	Asylah	JQ957057	-	JQ957345	JQ957133	JQ957262	JQ957422	-
H. homoeolepis	S7657	IBES7657	Oman	Asylah	JQ957057	JQ957195	JQ957343	JQ957133	JQ957262	JQ957422	-
H. homoeolepis	S7673	IBES7673	Oman	Asylah	JQ957057	-	JQ957344	JQ957133	JQ957262	JQ957422	-
H. homoeolepis	S7664	IBES7664	Oman	Asylah	JQ957057	JQ957195	JQ957344	JQ957133	JQ957262	JQ957422	-
H. homoeolepis	S7668	IBES7668	Oman	Asylah	JQ957057	JQ957195	JQ957344	JQ957133	JQ957262	JQ957422	-
H. homoeolepis	S7966	IBES7966	Oman	14.5 km NE of Sharbthat	JQ957057	JQ957197	JQ957350	JQ957136	JQ957262	JQ957422	-
H. homoeolepis	S7924	IBES7924	Oman	14.5 km NE of Sharbthat	JQ957057	JQ957197	JQ957348	JQ957135	JQ957262	JQ957422	-
H. homoeolepis	S7871		Oman	5 km W of Mughsayl	JQ957057	JQ957196	JQ957346	JQ957134	JQ957265	JQ957422	-
H. homoeolepis	AO81	IBEAO81	Oman	3 km SE of Haluf	JQ957057	JQ957191	JQ957340	JQ957131	JQ957262	JQ957422	-
H. homoeolepis	S7909	IBES7909	Oman	Wadi Ayoun	JQ957057	JQ957196	JQ957340	JQ957131	JQ957262	JQ957422	-
H. homoeolepis	AO85	IBEAO85	Oman	N of Wadi Ayoun	JQ957058	JQ957192	JQ957340	JQ957131	-	JQ957422	-
H. homoeolepis	AO119	IBEAO119	Oman	Wadi Ayoun	JQ957056	JQ957190	JQ957339	JQ957131	JQ957262	JQ957422	-
H. homoeolepis	S7893	IBES7893	Oman	5 km W of Mughsayl	JQ957060	JQ957196	JQ957347	-	JQ957266	JQ957422	-
H. homoeolepis	JS5	NMP6V 74805/1	Yemen	3 km E. Hauf	JQ957111	KC818832	-	-	-	-	-
H. homoeolepis	JS6	NMP6V 74805/2	Yemen	3 km E. Hauf	JQ957108	KC818832	-	KC818761	KC818916	JQ957422	KC818973
H. homoeolepis	JS8	NMP6V 74806	Yemen	Damqawt	JQ957109	KC818834	-	-	-	-	-
H. homoeolepis	JS75	NMP6V 74863	Oman	Mughsayl	JQ957112	KC818833	-	-	-	-	-
H. homoeolepis	IBES3807		Yemen	Wadi Ayahft, Socotra Island	JQ982793	JQ982908	JQ982725	KC818762	JQ982658	JQ957422	-
H. homoeolepis	IBES5142		Yemen	Cave Close Qualansia, Socotra Island	JQ982796	JQ982913	JQ982730	JQ957132	JQ982656	KC819034	-
H. homoeolepis	IBES5060		Yemen	Type Loc of H. dracaenacolus, Socotra	JQ982794	JQ982910	-	JQ957132	JQ982659	JQ957422	-
H. homoeolepis	IBES5154		Yemen	Darsa Island	JQ982795	JQ982914	JQ982729	JQ957132	JQ982656	JQ957422	-
H. homoeolepis	IBES5305		Yemen	Khaysat, Samhah Island	JQ982797	JQ982904	JQ982721	JQ957132	JQ982656	JQ957422	-
H. homoeolepis	IBES5106[#]		Yemen	Darsa Island	JQ982795	JQ982912	JQ982729	KC818763	JQ982656	JQ957422	-
H. homoeolepis	IBES5430		Yemen	W of Fikhah, Socotra Island	JQ982798	JQ982919	JQ982735	KC818764	JQ982656	JQ957422	-
H. inexpectatus	S1892	BM2008.712	Oman	2.5 km SE Ar Rumayliyah	JQ957066	JQ957206	JQ957364	JQ957141	JQ957274	JQ957427	-
H. inexpectatus	S7700	IBES7700	Oman	2.5 km SE Ar Rumayliyah	JQ957066	JQ957209	JQ957367	JQ957140	JQ957277	JQ957426	-
H. inexpectatus	S2166	BM2008.711	Oman	2.5 km SE Ar Rumayliyah	JQ957067	JQ957207	JQ957365	JQ957140	JQ957275	JQ957426	-
H. inexpectatus	S7735	IBES7735	Oman	2.5 km SE Ar Rumayliyah	JQ957067	JQ957210	JQ957368	JQ957140	JQ957273	JQ957426	-
H. inexpectatus	S1798[#]	S1798	Oman	2.5 km SE Ar Rumayliyah	JQ957065	JQ957205	JQ957363	JQ957140	JQ957273	JQ957426	-
H. inexpectatus	S7722	IBES7722	Oman	2.5 km SE Ar Rumayliyah	JQ957068	-	JQ957366	JQ957140	JQ957274	JQ957426	-
H. inexpectatus	S7674	IBES7674	Oman	2.5 km SE Ar Rumayliyah	JQ957067	JQ957208	JQ957366	JQ957140	JQ957276	JQ957426	-
H. inintellectus	IBES3290		Yemen	Wadi Kilisan S of Afafes Momi plateau,	JQ982799	JQ982924	JQ982738	-	JQ982666	-	-
H. inintellectus	IBES5212		Yemen	Close To Shuab, Socotra Island	JQ982804	JQ982925	JQ982750	KC818767	JQ982668	KC819035	-
H. inintellectus	IBES5068		Yemen	Wadi Ayahft, Socotra Island	JQ982802	JQ982922	JQ982748	KC818766	JQ982673	KC819035	-
H. inintellectus	IBES5102		Yemen	Wadi Ayahft, Socotra Island	JQ982802	JQ982929	JQ982737	KC818767	JQ982668	KC819035	-
H. inintellectus	IBES5110[#]		Yemen	Cave Close Qualansia, Socotra Island	JQ982803	JQ982930	JQ982742	KC818768	JQ982668	KC819035	-
H. inintellectus	IBE5439		Yemen	W of Fikhah, Socotra Island	JQ982806	JQ982931	JQ982749	KC818765	JQ982671	KC819035	-
H. inintellectus	IBE5431		Yemen	Lingohur Momi plateau, Socotra Island	JQ982805	JQ982932	JQ982751	KC818765	JQ982666	KC819035	-
H. inintellectus	IBE3782		Yemen	Homhil, Socotra Island	JQ982800	JQ982921	JQ982736	KC818765	JQ982671	KC819035	-
H. isolepis	JS202[#]		Somalia	8 km S of Borama	KC818680	KC818835	-	KC818769	KC818917	KC819036	KC818974
H. jumailiae	JS100	NMP6V 74819	Yemen	Sana´a	KC818681	KC818836	-	-	-	KC819037	-
H. jumailiae	JS53[#]	NMP6V 74818/1	Yemen	25 km NW of Al Bayda	KC818682	KC818837	-	KC818770	KC818918	KC819038	KC818975
H. jumailiae	JS54	NMP6V 74818/2	Yemen	25 km NW of Al Bayda	KC818682	KC818838	-	-	-	-	-
H. lavadeserticus	Hd31	NMP6V 74049/1	Syria	Ar´Raqiyeh	KC818684	HQ833742	-	-	-	-	-
H. lavadeserticus	Hd70[#]	NMP6V 74049/2	Syria	Ar´Raqiyeh	KC818683	HQ833743	-	JQ957161	KC818919	KC819039	KC818976
H. lavadeserticus	Hd74	NMP6V 74049/5	Syria	Ar´Raqiyeh	KC818684	HQ833746	-	-	-	-	-
H. lemurinus	AO117		Oman	Wadi Ayoun	JQ957062	JQ957200	JQ957352	JQ957137	JQ957268	JQ957423	-
H. lemurinus	AO123		Oman	Wadi Ayoun	JQ957062	JQ957200	JQ957354	JQ957137	-	JQ957423	-
H. lemurinus	AO116		Oman	Wadi Ayoun	JQ957061	JQ957199	JQ957351	JQ957137	JQ957267	-	-
H. lemurinus	AO118		Oman	Wadi Ayoun	JQ957061	JQ957201	JQ957353	JQ957137	JQ957269	JQ957423	-
H. lemurinus	AO124		Oman	Wadi Ayoun	JQ957061	JQ957201	JQ957355	JQ957137	-	JQ957423	-
H. lemurinus	JS9[#]		Yemen	Damqawt	JQ957113	KC818839	-	JQ957137	KC818921	JQ957423	KC818978
H. lemurinus	JS10	NMP6V 74801/1	Yemen	Damqawt	JQ957114	-	-	-	-	-	-
H. lemurinus	JS11	NMP6V 74801/2	Yemen	Damqawt	JQ957113	KC818839	-	JQ957137	KC818920	JQ957423	KC818977
H. luqueorum	S2152[#]	BM2005.1659	Oman	Wadi Bani Habib, Jebel Akhdar	JQ957070	JQ957214	JQ957372	JQ957146	JQ957278	JQ957431	-
H. luqueorum	AO46	BM2005.1660	Oman	Sayq, Jebel Akhdar	JQ957069	JQ957212	JQ957370	JQ957143	JQ957279	JQ957429	-
H. luqueorum	AO155	BM2005.1661	Oman	Sayq, Jebel Akhdar	JQ957069	JQ957211	JQ957369	JQ957142	JQ957278	JQ957428	-
H. luqueorum	AO59	BM2005.1658	Oman	Wadi Bani Habib, Jebel Akhdar	JQ957069	JQ957212	JQ957371	JQ957144	JQ957280	JQ957430	-
H. luqueorum	S8068	IBES8068	Oman	Wadi al Khahafa, Jebel Akhdar	JQ957069	JQ957215	JQ957373	JQ957147	JQ957281	JQ957430	-
H. luqueorum	S1756	BM2008.710	Oman	Wadi al Khahafa, Jebel Akhdar	JQ957069	JQ957213	-	JQ957145	JQ957281	JQ957430	-
H. mabouia	JS173	NMP6V 74804	Uganda	Mpanga forest	KC818685	KC818840	-	KC818771	KC818922	KC819040	KC818979
H. macropholis	CAS227511[#]	CAS227511	Somalia	11 km SE of Bosasso	DQ120379	DQ120208	JQ957356	JQ957138	JQ957270	JQ957424	-
H. masirahensis	S3412		Oman	Wadi Harf, Masirah island	JQ957063	JQ957202	JQ957359	JQ957139	JQ957272	JQ957425	-
H. masirahensis	S7707	IBES7707	Oman	Wadi Maahdi, Masirah island	JQ957063	JQ957202	JQ957361	JQ957139	JQ957272	JQ957425	-
H. masirahensis	S1878	BM2008.713	Oman	Wadi Haql. Masirah island	JQ957063	JQ957202	JQ957357	JQ957139	-	JQ957425	-
H. masirahensis	S7661	IBES7661	Oman	Wadi Maahdi, Masirah island	JQ957063	JQ957202	-	JQ957139	JQ957272	JQ957425	-
H. masirahensis	S2044[#]	S2044	Oman	Wadi Haql, Masirah island	JQ957064	JQ957203	JQ957358	JQ957139	JQ957271	JQ957425	-
H. masirahensis	S7651	IBES7651	Oman	Wadi Maahdi, Masirah island	JQ957063	JQ957204	JQ957360	JQ957139	JQ957272	JQ957425	-
H. masirahensis	S7710	IBES7710	Oman	Wadi Maahdi, Masirah island	JQ957063	JQ957204	JQ957362	JQ957139	JQ957272	JQ957425	-
H. mindiae	Hd22[#]	NMP6V 72323/2	Jordan	Jabal Ghazali	KC818686	HQ833747	-	JQ957128	-	JQ957423	KC818980
H. mindiae	Hd23	NMP6V 72739/1	Jordan	Wadi Ramm Nughra	KC818686	HQ833748	-	JQ957128	-	KC819041	KC818981
H. modestus	CAS198934[#]	CAS198934	Kenya	Kijiado District, Rift Valley Province	DQ120386	DQ120215	-	JQ957149	JQ957283	JQ957432	-
H. ophiolepis	JS215[#]		Ethiopia	15 km NE of Dire Dawa	KC818689	KC818841	-	KC818772	KC818923	KC819042	KC818982
H. oxyrhinus	IBE2624[#]		Yemen	Bir Al Aguz ,Abd al Kuri Island	JQ982807	JQ982936	JQ982754	KC818773	JQ982676	JQ957425	-
H. oxyrhinus	IBE5394		Yemen	Bir Al Aguz ,Abd al Kuri Island	JQ982810	JQ982935	JQ982757	KC818773	JQ982677	JQ957425	-
H. oxyrhinus	IBE3002		Yemen	Bir Al Aguz ,Abd al Kuri Island	JQ982808	JQ982937	JQ982755	KC818774	JQ982678	KC819043	-
H. oxyrhinus	IBE5435		Yemen	Bir Al Aguz ,Abd al Kuri Island	JQ982811	JQ982941	JQ982760	KC818774	JQ982677	JQ957425	-

SPECIES	CODE	VOUCHER	COUNTRY	LOCALITY	*12S*	*cytb*	ND4 + tRNAs	*cmos*	*mc1r*	*rag2*	*rag1*
H. oxyrhinus	IBE4058		Yemen	Bir Al Aguz ,Abd al Kuri Island	JQ982807	JQ982940	JQ982759	KC818773	JQ982676	JQ957425	-
H. oxyrhinus	IBE5382		Yemen	Khaysat Salih ,Abd al Kuri Island	JQ982809	JQ982942	JQ982764	KC818774	JQ982675	JQ957425	-
H. paucituberculatus	S7988	IBES7988	Oman	Khor Sauli	JQ957072	-	JQ957383	JQ957150	JQ957291	JQ957425	-
H. paucituberculatus	AO104[#]	IBEAO104	Oman	East Khor	JQ957072	JQ957217	JQ957375	JQ957150	JQ957284	JQ957425	-
H. paucituberculatus	AO162		Oman	Khor Sauli	JQ957072	JQ957217	JQ957376	JQ957151	JQ957285	JQ957425	-
H. paucituberculatus	S3261		Oman	Wadi Darbat	JQ957072	JQ957217	JQ957379	JQ957150	JQ957284	-	-
H. paucituberculatus	S7921	IBES7921	Oman	Khor Sauli	JQ957072	JQ957217	JQ957376	JQ957150	JQ957289	-	-
H. paucituberculatus	S7646	IBES7646	Oman	Khor Sauli	JQ957072	JQ957217	JQ957380	JQ957150	JQ957287	JQ957425	-
H. paucituberculatus	S7910	IBES7910	Oman	East Khor	JQ957072	JQ957217	JQ957377	JQ957150	JQ957286	JQ957425	-
H. paucituberculatus	AO91	IBEAO91	Oman	East Khor	JQ957072	JQ957217	JQ957377	JQ957151	-	JQ957425	-
H. paucituberculatus	S7994	IBES7994	Oman	Wadi Darbat	JQ957072	JQ957221	JQ957384	JQ957151	JQ957292	JQ957425	-
H. paucituberculatus	S3235		Oman	Wadi Darbat	JQ957072	JQ957218	JQ957378	JQ957150	JQ957286	-	-
H. paucituberculatus	S8004	IBES8004	Oman	3.5 km NE Sadah	JQ957072	JQ957222	JQ957385	JQ957150	JQ957287	JQ957425	-
H. paucituberculatus	S7930	IBES7930	Oman	Wadi Hasik	JQ957073	JQ957220	JQ957382	JQ957150	JQ957290	JQ957433	-
H. paucituberculatus	S7902	IBES7902	Oman	3 km NW of Hasik	JQ957073	JQ957219	-	JQ957150	JQ957288	JQ957425	-
H. paucituberculatus	S7812		Oman	Wadi Hasik	JQ957073	JQ957219	JQ957381	JQ957150	JQ957288	-	-
H. persicus	FTHM005100	FTHM005100	Iran	Bushehr	JQ957076	JQ957224	-	JQ957152	-	-	-
H. persicus	MVZ234385[#]	MVZ Herps	Iran	Lipar Village, Sistan and Baluchistan	JQ957077	JQ957225	JQ957386	JQ957152	JQ957293	JQ957435	-
H. persicus	FTHM005000	FTHM005000	Iran	Mahshar	JQ957074	JQ957223	-	JQ957152	-	JQ957434	-
H. persicus	FTHM005001	FTHM005001	Iran	Mahshar	JQ957075	JQ957223	-	JQ957152	-	-	-
H. persicus	JS103[#]	NMP6V 74807/1	Iran	Booreki	KC818691	KC818842	-	KC818775	KC818924	KC819044	KC818983
H. persicus	JS104	NMP6V 74807/2	Iran	Booreki	KC818690	KC818843	-	KC818775	KC818925	JQ957434	KC818984
H. persicus	JS105	NMP6V 74807/3	Iran	Booreki	KC818691	KC818844	-	-	-	-	-
H. platycephalus	JS168	NMP6V 74864/1	Kenya	Ngurunit	KC818692	-	-	-	-	-	-
H. platycephalus	JS169	NMP6V 74864/2	Kenya	Ngurunit	KC818693	KC818845	-	KC818776	KC818926	KC819045	KC818985
H. platycephalus	JS171	NMP6V 74865/1	Kenya	Wamba	KC818694	KC818846	-	KC818776	KC818926	KC819046	KC818986
H. platycephalus	JS172	NMP6V 74865/2	Kenya	Wamba	KC818695	-	-	-	-	-	-
H. pumilio	IBES3307		Yemen	Near Qedami, Socotra Island	JQ982813	JQ982946	JQ982769	KC818777	JQ982685	KC819047	-
H. pumilio	IBES3382		Yemen	Damir Wadi Luntir, Socotra Island	JQ982815	JQ982951	JQ982774	-	JQ982689	-	-
H. pumilio	IBES3404		Yemen	Ghubbat Timbar, Socotra Island	JQ982816	JQ982952	JQ982770	KC818777	JQ982690	KC819048	-
H. pumilio	IBES3341		Yemen	Between Jooh and Di Ishal, Socotra Island	JQ982814	JQ982949	JQ982772	-	JQ982683	-	-
H. pumilio	IBES5272		Yemen	Socotra Island	JQ982813	JQ982955	JQ982777	KC818777	JQ982694	KC819049	-
H. pumilio	IBES5117[#]		Yemen	Ridah, Socotra Island	JQ982818	JQ982954	JQ982776	KC818777	JQ982683	KC819047	-
H. pumilio	IBES5021		Yemen	Steroh, Socotra Island	JQ982817	JQ982948	JQ982771	KC818777	JQ982693	KC819047	-
H. pumilio	IBES5616		Yemen	Firmin, Socotra Island	JQ982820	JQ982957	JQ982779	KC818777	JQ982699	KC819050	-
H. pumilio	IBES5658		Yemen	South, Socotra Island	JQ982822	JQ982961	JQ982780	KC818777	JQ982683	KC819047	-
H. pumilio	IBES5402		Yemen	Tahr Diksam, Socotra Island	JQ982819	JQ982956	JQ982778	KC818777	JQ982697	KC819047	-
H. robustus	S2151		Oman	8 km W Shannah	JQ957081	JQ957228	JQ957389	JQ957158	JQ957294	JQ957409	-
H. robustus	S1677		Oman	1 km W airport, Masirah Island	JQ957080	JQ957228	JQ957389	JQ957153	-	JQ957409	-
H. robustus	S1905		Oman	1 km W airport, Masirah Island	JQ957080	JQ957228	JQ957389	JQ957158	JQ957294	JQ957438	-
H. robustus	S1962		Oman	1 km W airport, Masirah Island	JQ957080	JQ957228	JQ957389	JQ957154	JQ957294	JQ957409	-
H. robustus	S1788		Oman	1 km W airport, Masirah Island	JQ957080	JQ957228	JQ957389	JQ957154	JQ957294	JQ957436	-
H. robustus	S2150		Oman	1 km W airport, Masirah Island	JQ957080	JQ957228	JQ957391	JQ957159	-	JQ957409	-
H. robustus	SPM001859		Egypt	Safaga	DQ120347	DQ120176	JQ957394	JQ957160	JQ957298	JQ957439	-
H. robustus	AO164b		Oman	East Khor	JQ957078	JQ957226	JQ957387	JQ957153	JQ957294	JQ957436	-
H. robustus	AO165		Oman	East Khor	JQ957078	JQ957226	JQ957387	JQ957154	JQ957295	JQ957437	-
H. robustus	AO4		Oman	Al Azaiba	JQ957079	JQ957227	-	-	-	-	-
H. robustus	UAE25		UAE	Wadi Tayybiyah	JQ957079	JQ957227	-	-	-	-	-
H. robustus	AO3		Oman	Al Azaiba	JQ957079	JQ957227	JQ957388	JQ957155	JQ957294	JQ957409	-
H. robustus	Hturc2		UAE	Dhafra Beach, near Ruwais	AF186117	AF184989	-	JQ957156	-	JQ957409	-
H. robustus	SPM001501		UAE	Dhafra Beach, near Ruwais	AF186117	JQ957227	-	-	-	-	-
H. robustus	R1415		Yemen	Mukalla Airport	AF186117	JQ957229	JQ957392	JQ957154	JQ957297	JQ957436	-
H. robustus	S1688		Oman	8 km W Shannah	AF186117	JQ957226	JQ957390	JQ957157	JQ957296	JQ957409	-
H. robustus	SPM001503		UAE	Abu Dhabi	AF186117	JQ957226	JQ957393	-	-	-	-
H. robustus	JS50[#]	NMP6V 74821/1	Yemen	Wadi Zabid	KC818701	KC818852	-	KC818779	KC818928	JQ957409	KC818987
H. robustus	JS51	NMP6V 74821/2	Yemen	Wadi Zabid	KC818701	KC818853	-	-	-	-	-
H. robustus	JS58[#]	NMP6V 74829	Yemen	Bir Ali	KC818702	KC818854	-	KC818780	KC818929	KC819051	KC818988
H. robustus	JS66	NMP6V 74867/1	Oman	Muscat Airport	KC818701	KC818855	-	-	-	-	-
H. robustus	JS67	NMP6V 74867/2	Oman	Muscat Airport	KC818703	KC818849	-	KC818781	KC818930	JQ957409	KC818989
H. robustus	JS68	NMP6V 74867/3	Oman	Muscat Airport	KC818701	KC818856	-	-	-	-	-
H. robustus	JS69	NMP6V 74868	Oman	Salalah	KC818699	KC818857	-	-	-	-	-
H. robustus	JS74	NMP6V 73869/1	Oman	Mughsayl	KC818699	KC818850	-	-	-	-	-
H. robustus	JS76	NMP6V 74869/2	Oman	Mughsayl	KC818699	KC818850	-	-	-	-	-
H. robustus	JS82		Oman	Al Qarbi	KC818701	KC818858	-	-	-	-	-
H. robustus	JS83	NMP6V 74870/1	Oman	Shisr	KC818704	KC818859	-	-	-	-	-
H. robustus	JS84	NMP6V 74870/2	Oman	Shisr	KC818705	KC818859	-	-	-	-	-
H. robustus	JS101		Somalia	Berbera	KC818696	KC818847	-	KC818778	KC818927	JQ957409	KC818987
H. robustus	JS102		Somalia	Berbera	KC818697	KC818848	-	-	-	-	-
H. robustus	JS106	NMP6V 74820	Iran	Bandar-e Lengeh	KC818698	KC818849	-	-	-	-	-
H. robustus	JS120	NMP6V 74869/3	Oman	Mughsayl	KC818699	KC818850	-	-	-	-	-
H. robustus	JS210		Ethiopia	Jijiga	KC818700	KC818851	-	-	-	-	-
H. ruspolii	JS177	NMP6V 74871/3	Kenya	Kalacha	KC818706	KC818860	-	KC818782	KC818931	KC819052	-
H. ruspolii	JS192	NMP6V 74871/1	Kenya	Kalacha	KC818706	-	-	-	-	-	-
H. ruspolii	JS193	NMP6V 74871/2	Kenya	Kalacha	KC818706	-	-	KC818782	-	-	-
H. shihraensis	JS16[#]	NMP6V 74816	Yemen	11 km N of Mukalla	KC818710	-	-	KC818783	KC818932	KC819053	KC818990
H. shihraensis	JS55	NMP6V 74817/1	Yemen	Ghayl Ba Wazir	KC818707	KC818862	-	-	-	-	-
H. shihraensis	JS56	NMP6V 74817/2	Yemen	Ghayl Ba Wazir	KC818711	KC818864	-	KC818784	KC818933	KC819054	KC818991
H. shihraensis	JS57	NMP6V 74817/3	Yemen	Ghayl Ba Wazir	KC818707	KC818865	-	KC818783	KC818934	JQ957410	KC818992
H. shihraensis	JS133	NMP6V 74817/4	Yemen	Ghayl Ba Wazir	KC818707	KC818861	-	-	-	-	-
H. shihraensis	JS134	NMP6V 74817/5	Yemen	Ghayl Ba Wazir	KC818707	KC818862	-	-	-	-	-
H. shihraensis	JS135	NMP6V 74817/6	Yemen	Ghayl Ba Wazir	KC818708	-	-	-	-	-	-
H. shihraensis	JS136	NMP6V 74817/7	Yemen	Ghayl Ba Wazir	KC818709	KC818863	-	-	-	-	-
H. shihraensis	JS137	NMP6V 74817/8	Yemen	Ghayl Ba Wazir	KC818707	KC818862	-	-	-	-	-
H. shihraensis	JS138	NMP6V 74817/9	Yemen	Ghayl Ba Wazir	KC818707	KC818862	-	-	-	-	-
H. shihraensis	Hd90	NMP6V 74169	Yemen	Ghayl Ba Wazir	-	HQ833760	-	-	-	-	-
H. sinaitus	JS146	NMP6V 74809/1	Sudan	Wad Ben Naga	KC818712	KC818866	-	JQ957164	KC818935	JQ957446	KC818993
H. sinaitus	JS147	NMP6V 74809/2	Sudan	Wad Ben Naga	KC818713	KC818867	-	JQ957164	JQ957302	JQ957446	KC818994
H. sinaitus	JS148	NMP6V 74809/3	Sudan	Wad Ben Naga	KC818712	KC818868	-	-	-	-	-
H. sinaitus	JS149	NMP6V 74809/4	Sudan	Wad Ben Naga	KC818714	KC818869	-	-	-	-	-
H. sinaitus	JS150[#]	NMP6V 74810	Sudan	15 km SE of Atbara	KC818712	KC818869	-	JQ957164	JQ957303	JQ957446	KC818995
H. smithi	JS208		Somalia	30 km N of Shiikh	KC818715	KC818870	-	KC818785	KC818936	KC819055	KC818996
H. squamulatus	JS160	NMP6V 74872/2	Kenya	South Horr	KC818737	-	-	-	-	-	-
H. squamulatus	JS162	NMP6V 74872/4	Kenya	South Horr	KC818737	-	-	-	-	-	-
H. squamulatus	JS180		Kenya	South Horr	KC818738	-	-	-	-	-	-
H. squamulatus	JS183[#]		Kenya	Isiolo	KC818739	-	-	JQ957149	KC818946	KC819065	KC819005
H. squamulatus	JS190	NMP6V 74872/5	Kenya	South Horr	KC818737	-	-	-	-	-	-
H. squamulatus	JS191[#]	NMP6V 74872/6	Kenya	South Horr	KC818737	KC818888	-	JQ957149	KC818947	KC819066	KC819006
H. turcicus	SPM000788		Turkey	Erzin	DQ120334	DQ120163	JQ957398	JQ957162	-	JQ957443	-
H. turcicus	SPM001629		Spain	Torregorda, Cádiz	DQ120311	DQ120140	JQ957399	JQ957162	JQ957301	JQ957444	-
H. turcicus	SPM002086		Spain	Barcelona	DQ120313	DQ120142	JQ957400	JQ957163	JQ957301	JQ957445	-
H. turcicus	Hd55		Albania	Ardenica	KC818742	HQ833711	-	JQ957162	KC818949	KC819068	KC819009
H. turcicus	Hd62	NMP6V 73626/1	Turkey	Finike	KC818743	HQ833736	-	-	-	-	-
H. turcicus	Hd66		Croatia	Sumartin	KC818742	HQ833714	-	-	-	-	-
H. turcicus	JS152	NMP6V 74215	Lebanon	Aamchit	KC818742	HQ833714	-	JQ957162	KC818950	KC819069	KC819010
H. turcicus	Hd2[#]		Israel	Almagor	KC818740	HQ833741	-	KC818793	KC818948	JQ957423	KC819007
H. turcicus	Hd5		Turkey	Adana	KC818741	HQ833735	-	JQ957162	-	JQ957423	-
H. turcicus	Hd34[#]		Egypt	Sharn All Shiekh	KC818741	HQ833717	-	JQ957162	JQ957301	KC819067	KC819008
H. turcicus	Hd37	NMP6V 72081	Egypt	El Arish	KC818741	HQ833718	-	-	-	-	-
H. yerburii yerburii	JS40[#]	NMP6V 74824/1	Yemen	3 km S of Najd an Nashamah	JQ957086	KC818894	-	JQ957167	JQ957306	JQ957447	KC819013

SPECIES	CODE	VOUCHER	COUNTRY	LOCALITY	*12S*	*cytb*	ND4 + tRNAs	*cmos*	*mc1r*	*rag2*	*rag1*
H. yerburii yerburii	JS44	NMP6V 74828/2	Yemen	Al Hababi	JQ957086	KC818897	-	JQ957166	JQ957307	JQ957448	KC819014
H. yerburii yerburii	JS60	NMP6V 74822/2	Yemen	6 km N of Al Hisn	JQ957087	KC818893	-	JQ957166	JQ957308	JQ957447	KC819015
H. yerburii yerburii	JS29#	NMP6V 74826	Yemen	8 km N of Lahij	JQ957085	KC818892	-	JQ957165	JQ957304	JQ957447	KC819011
H. yerburii yerburii	JS30	-	Yemen	8 km N of Lahij	JQ957085	KC818893	-	JQ957166	JQ957305	JQ957447	KC819012
H. yerburii yerburii	JS20	NMP6V 74168	Yemen	27 km S of Taizz	KC818744	HQ833762	-	-	-	-	-
H. yerburii yerburii	JS23	NMP6V 74827/2	Yemen	Jabel Habeshi	JQ957086	KC818889	-	-	-	-	-
H. yerburii yerburii	JS24	NMP6V 74825/1	Yemen	At Turbah	JQ957086	KC818890	-	-	-	-	-
H. yerburii yerburii	JS26	NMP6V 74825/2	Yemen	At Turbah	JQ957086	KC818891	-	-	-	-	-
H. yerburii yerburii	JS33	NMP6V 74823/1	Yemen	14 km NW of At Turbah	JQ957086	KC818894	-	-	-	-	-
H. yerburii yerburii	JS34	NMP6V 74823/2	Yemen	14 km NW of At Turbah	JQ957086	KC818894	-	-	-	-	-
H. yerburii yerburii	JS35	NMP6V 74823/3	Yemen	14 km NW of At Turbah	JQ957086	KC818894	-	-	-	-	-
H. yerburii yerburii	JS41	NMP6V 74824/2	Yemen	3 km S of Najd an Nashamah	JQ957086	KC818894	-	-	-	-	-
H. yerburii yerburii	JS42	NMP6V 74828/1	Yemen	Al Hababi	JQ957086	KC818895	-	-	-	-	-
H. yerburii yerburii	JS43	-	Yemen	Al Hababi	JQ957086	KC818896	-	-	-	-	-
H. yerburii yerburii	JS61	NMP6V 74822/3	Yemen	6 km N of Al Hisn	JQ957087	KC818898	-	-	-	-	-
H. yerburii montanus	JS28	NMP6V 74802	Yemen	Jabal Bura	KC818687	KC818899	-	-	-	-	-
H. yerburii montanus	JS52#	NMP6V 74803	Yemen	5 km NE of Hajjah	KC818688	KC818900	-	KC818750	KC818951	KC819070	KC819016
H. sp. 1	Hd41	NMP6V70163/2	Egypt	Sharm All Shiekh	KC818724	HQ833759	-	-	-	-	KC818981
H. sp. 1	Sher10660#	Sher10660	Egypt	Ayoun Musa	JQ957071	JQ957216	JQ957374	JQ957148	JQ957282	JQ957409	-
H. sp. 4	JS17#	NMP6V 74831/1	Yemen	Al Hadr	KC818725	KC818874	-	KC818787	KC818940	KC819059	KC818999
H. sp. 4	JS18	NMP6V 74831/2	Yemen	Al Hadr	KC818725	-	-	-	-	-	-
H. sp. 4	JS32	NMP6V 74835	Yemen	35 km W of Lahij	KC818726	KC818875	-	KC818788	KC818941	KC819060	KC819000
H. sp. 4	JS37	NMP6V 74832/1	Yemen	3 km S of Najd an Nashamah	KC818727	KC818876	-	-	-	-	-
H. sp. 4	JS38	NMP6V 74832/2	Yemen	3 km S of Najd an Nashamah	KC818727	KC818877	-	-	-	-	-
H. sp. 4	JS45		Yemen	Al Hababi	KC818728	KC818878	-	-	-	-	-
H. sp. 4	JS46	NMP6V 74833/1	Yemen	Al Hababi	KC818728	KC818879	-	-	-	-	-
H. sp. 4	JS47	NMP6V 74833/2	Yemen	Al Hababi	KC818729	KC818880	-	KC818789	KC818942	KC819061	KC819001
H. sp. 4	JS48	NMP6V 74834/1	Yemen	Wadi Zabid	KC818730	KC818881	-	KC818789	KC818943	KC819062	KC819001
H. sp. 4	JS49	NMP6V 74834/2	Yemen	Wadi Zabid	KC818731	KC818882	-	-	-	-	-
H. sp. 5	JS27		Yemen	Jabal Sabir	KC818732	KC818883	-	-	-	-	-
H. sp. 5	JS36#	NMP6V 74836/1	Yemen	3 km S of Najd an Nashamah	KC818734	KC818884	-	KC818790	-	JQ957409	KC819002
H. sp. 5	JS39	NMP6V 74836/2	Yemen	3 km S of Najd an Nashamah	KC818733	KC818885	-	-	-	-	-
H. sp. 6	JS31#	NMP6V 74837	Yemen	8 km N of Lahij	KC818735	KC818886	-	KC818791	KC818944	KC819063	KC819003
H. sp. 9	JS216#		Ethiopia	10 km E of Yidi	KC818736	KC818887	-	KC818792	KC818945	KC819064	KC819004
H. sp. 9	JS217		Ethiopia	Awash	KC818736	KC818887	-	-	-	-	-
H. sp. 10	JS181#		Kenya	Gus	KC818716	KC818871	-	KC818769	KC818937	KC819056	KC818997
H. sp. 10	JS182		Kenya		KC818717	-	-	-	-	-	-
H. sp. 10	JS184#		Kenya	Kalacha	KC818718	KC818872	-	KC818769	KC818938	KC819057	-
H. sp. 10	JS185		Kenya	Kalacha	KC818719	-	-	-	-	-	-
H. sp. 10	JS186		Kenya	Kalacha	KC818719	-	-	-	-	-	-
H. sp. 10	JS187		Kenya		KC818720	-	-	KC818769	-	-	-
H. sp. 10	JS188		Kenya		KC818721	-	-	-	-	-	-
H. sp. 10	JS189		Kenya		KC818721	-	-	-	-	-	-
H. sp. 10	JS194		Kenya	Korante plain	KC818721	-	-	-	-	-	-
H. sp. 10	JS195		Kenya	Korante plain	KC818722	-	-	-	-	-	-
H. sp. 11	JS212#		Ethiopia	2 km N of Metehara	KC818723	KC818873	-	KC818786	KC818939	KC819058	KC818998
