# Supplementary material for: Out of Arabia: A Complex Biogeographic History of Multiple Vicariance and Dispersal Events in the Gecko Genus Hemidactylus (Reptilia: Gekkonidae)
Source: PLoS One. 2013 May 27;8(5):e64018. doi: 10.1371/journal.pone.0064018 (PMC3664631; doi:10.1371/journal.pone.0064018)
Supplement: Table S2 — Molecular markers, primers, primer sequences, amplification conditions and original primer sources used in this study. (PDF) [file pone.0064018.s007.pdf]

Table S2. Molecular markers, primers, primer sequences, amplification conditions and original primer sources used in this study

Gene	Primer name	Primer sequence (5´- 3´)	Analyzed fragment length (bp)	T^a annealing	Primer source
12S rRNA	12Sa	AAACTGGGATTAGATACCCCACTAT	381 - 396	48º	Kocher et al. 1989
	12Sb	TGAGGAGGGTGACGGGCGGT			
cytb short	Cytb1	CCATCCAACATCTCAGCATGATGAAA	307	46º	Kocher et al. 1989 (shortened)
	Cytb2	CCCTCAGAATGATATTTGTCCTCA			
cytb long	L14910	GACCTGTGATMTGAAAACCAYCGTTGT	1137	46º	Burbrink et al. 2000
	H16064	CTTTGGTTTACAAGAACAATGCTTTA			
cmos	FUF	TTTGGTTCKGTCTACAAGGCTAC	403	53º	Gamble et al. 2008
	FUR	AGGGAACATCCAAAGTCTCCAAT			
mc1r	MC1RF	AGGCNGCCATYGTCAAGAACCGGAACC	668	56°	Pinho et al. 2009
	MC1RR	CTCCGRAAGGCRTAAATGATGGGGTCCAC			
rag1 short	F700	GGAGACATGGACACAATCCATCCTAC	280	53º	Bauer et al. 2007
	R700	TTTGTACTGAGATGGATCTTTTTGCA			
rag1 long	R13	TCTGAATGGAAATTCAAGCTGTT	1023	58°	Groth and Barrowclough 1999
	R18	GATGCTGCCTCGGTCGGCCACCTTT			
rag2	Py1F	CCCTGAGTTTGGATGCTGTACTT	410	53º	Gamble et al. 2008
	Py1R	AACTGCCTRTTGTCCCCTGGTAT			

References to Table S2.

Bauer AM, DeSilva A, Greenbaum E, Jackman TR (2007) A new species of day gecko from high elevation in Sri Lanka, with a preliminary phylogeny of Sri Lankan *Cnemaspis* (Reptilia: Squamata: Gekkonidae). Mitt Mus Naturk Berlin Zool Reihe 83: 22-32.

Burbrink FT, Lawson R, Slowinski JB (2000) Mitochondrial DNA phylogeography of the polytypic North American rat snake (*Elaphe obsoleta*): A critique of the subspecies concept. Evolution 54: 2107–2118.

Gamble T, Bauer AM, Greenbaum E, Jackman TR (2008) Evidence for Gondwanan vicariance in an ancient clade of gecko lizards. J Biogeogr 35(1): 88-104.

Groth JG, Barrowclough GF (1999) Basal Divergences in Birds and the Phylogenetic Utility of the Nuclear RAG-1 Gene. Mol Phylogenet Evol 12(2): 115-123.

Kocher TD, Thomas WK, Meyer A, Edwards SV, Pääbo S et al. (1989) Dynamics of mitochondrial DNA evolution in animals: amplification and sequencing with conserved primers. Proc Natl Acad Sci USA 86: 6196–6200.

Pinho C, Rocha S, Carvalho BM, Lopes S, Mourao S et al. (2009) New primers for the amplification and sequencing of nuclear loci in a taxonomically wide set of reptiles and amphibians. Conserv Genet Resour 2(1): 181-185.
