# Supplementary material for: Out of Arabia: A Complex Biogeographic History of Multiple Vicariance and Dispersal Events in the Gecko Genus Hemidactylus (Reptilia: Gekkonidae)
Source: PLoS One. 2013 May 27;8(5):e64018. doi: 10.1371/journal.pone.0064018 (PMC3664631; doi:10.1371/journal.pone.0064018)
Supplement: Table S3 — List of all Hemidactylus species from Arabia, the Horn of Africa, the Levant and Iran. Black dots indicate known distribution records for each country, the rightmost column shows species included in this study. (PDF) [file pone.0064018.s008.pdf]

Table S3. List of all *Hemidactylus* species from Arabia, the Horn of Africa, the Levant and Iran. Black dots indicate known distribution records for each country, the rightmost column shows species included in this study.

	**Arabia**					**Iran**	**Africa**							**The Levant**				Used in this study
	Oman	Saudi Arabia	UAE	Yemen	Socotra Archipel.		Djibouti	Egypt	Eritrea	Ethiopia	Kenya	Somalia	Sudan	Israel	Jordan	Syria	Lebanon	
H. albopunctatus										●		●						●
H. alkiyumii	●			●														●
H. angulatus									●	●	●		●					●
H. arnoldi							●					●						
H. barbierii											●							
H. barodanus									●	●		●						●
H. bavazzanoi										●	●	●						
H. citernii												●						●
H. curlei							**?**			●		●						
H. dawudazraqi															●	●		●
H. dracaenacolus					●													●
H. endophis	●																	
H. festivus	●			●														●
H. flaviviridis	●	●	●	●	●	●	●	●	●	●		●	●					●
H. forbesii					●													●
H. foudaii								●					●					●
H. frenatus							●				●	●						
H. funaiolii											●	●						●
H. granchii												●						●
H. granti					●													●
H. hajarensis	●																	●
H. homoeolepis	●	●		●	●													●
H. inexpectatus	●																	●
H. inintellectus					●													●
H. isolepis										●	●	●						●
H. jubensis										●								
H. jumailiae				●														●
H. klauberi												●						
H. laevis												●						
H. laticaudatus									●	●								
H. lavadeserticus																●		●

H. lemurinus	●			●														●
H. leschenaultii	●																	
H. luqueorum	●																	●
H. mabouia									●	●	●	●	●					●
H. macropholis									?	●	●	●						●
H. masirahensis	●																	●
H. megalops												●						
H. mercatorius											●	●						
H. mindiae								●							●			●
H. modestus											●	●						●
H. ophiolepis										●								●
H. ophiolepoides										●		●						
H. oxyrhinus					●													●
H. paucituberculatus	●																	●
H. persicus	?	●	●			●												●
H. platycephalus										●	●	●						●
H. puccionii												●						
H. pumilio					●													●
H. robustus	●	●	●	●	●	●	●	●	●	●	●	●	●					●
H. romeshkanicus						●												
H. ruspolii										●	●	●						●
H. saba				●														●
H. shihraensis				●														●
H. sinaitus									●	●		●	●					●
H. smithi										●		●						●
H. somalicus										●		●						
H. squamulatus										●	●	●						●
H. taylori												●						
H. tropidolepis										●	●	●						
H. turcicus								●					●	●	●	●	●	●
H. yerburii yerburii				●														●
H. yerburii montanus				●														●
H. yerburii pauciporosus												●						
